# DNA barcoding for the identification and authentication of medicinal deer (*Cervus* sp.) products in China

**DOI:** 10.1371/journal.pone.0297164

**Published:** 2024-01-19

**Authors:** Wenlan Li, Qiqi Ren, Jian Feng, Shiou Yih Lee, Yangyang Liu

**Affiliations:** 1 School of Pharmacy, Harbin University of Commerce, Harbin, China; 2 Hainan Branch of the Institute of Medicinal Plant Development, Chinese Academy of Medical Sciences, Haikou, China; 3 Faculty of Health and Life Sciences, INTI International University, Nilai, Malaysia; University of Otago, NEW ZEALAND

## Abstract

Deer products from sika deer (*Cervus nippon*) and red deer (*C*. *elaphus*) are considered genuine and used for Traditional Chinese Medicine (TCM) materials in China. Deer has a very high economic and ornamental value, resulting in the formation of a characteristic deer industry in the prescription preparation of traditional Chinese medicine, health food, cosmetics, and other areas of development and utilization. Due to the high demand for deer products, the products are expensive and have limited production, but the legal use of deer is limited to only two species of sika deer and red deer; other wild deer are prohibited from hunting, so there are numerous cases of mixing and adulteration of counterfeit products and so on. There have been many reports that other animal (pig, cow, sheep, etc.) tissues or organs are often used for adulteration and confusion, resulting in poor efficacy of deer traditional medicine and trade fraud in deer products. To authenticate the deer products in a rapid and effective manner, the analysis used 22 deer products (antler, meat, bone, fetus, penis, tail, skin, and wool) that were in the form of blind samples. Total DNA extraction using a modified protocol successfully yielded DNA from the blind samples that was useful for PCR. Three candidate DNA barcoding loci, cox1, Cyt b, and rrn12, were evaluated for their discrimination strength through BLAST and phylogenetic clustering analyses. For the BLAST analysis, the 22 blind samples obtained 100% match identity across the three gene loci tested. It was revealed that 12 blind samples were correctly labeled for their species of origin, while three blind samples that were thought to originate from red deer were identified as *C*. *nippon*, and seven blind samples that were thought to originate from sika deer were identified as *C*. *elaphus*, *Dama dama*, and *Rangifer tarandus*. DNA barcoding analysis showed that all three gene loci were able to distinguish the two *Cervus* species and to identify the presence of adulterant species. The DNA barcoding technique was able to provide a useful and sensitive approach in identifying the species of origin in deer products.

## Introduction

Cervidae, which is more commonly known as the deer family, is the second-most diverse group of ruminants and is widely distributed all over the world [1]. At present, a total of 18 genera and more than 40 species have been recorded, with members of *Cervus* being one of the important genera that has been domesticated and introduced to other nonnative countries as park animals and food sources [2,3]. To date, a few countries have established the full domestication of deer for farming, which is commercially important in providing deer meat (venison) to an international market, velvet antler for the oriental medicine trade, and other less important products such as hides, pizzles, ligaments, and teeth [4].

There are at least five species of *Cervus* that are recorded in China, including *C*. *albirostris* (Thorold’s deer), *C*. *canadensis* (elk), *C*. *elaphus* (red deer), *C*. *eldii* (Eld’s deer), and *C*. *nippon* (sika deer) [5]. In the past, most of these deer species were used for medicinal purposes; various tissues and organs of deer, including the fetus, penis, tail, heart, meat, blood, and bone, were used in various applications in traditional medicine production, supplementary diet, wine production, etc. [6]. Health-benefiting features from deer products were also proven legitimate through scientific approaches [7–9]. As demand for deer products has seemingly increased over the years, overhunting and the loss of natural habitat due to urbanization have threatened the survival of these species in the wild. To cater to the demand for deer products, the Chinese government has promoted deer farming and stipulated that only farmed red deer and sika deer can be used for food and medicinal purposes; wild deer are generally protected animals by local enforcement agencies, and trades involving these wild deer are prohibited. Consequently, only deer antlers from red deer and sika deer are recognized as sources of traditional Chinese medicine (TCM) [10]. Because deer products are expensive and limited, cases of dishonest traders involved in adulterating deer products using body parts from pigs, cattle, and sheep have been reported [11,12]. As China is one of the main producers of deer products for both the local and international markets, it is important to ensure that trade fraud involving deer products is resolved due to food safety issues.

In general, identification of the source of origin in deer products greatly relies on the morphological characteristics [13] coupled with microscopic [14] and chemical analyses [15]. Although species identification of deer products can be accurate based on these methods, experienced or trained personnel are required to ensure that the identification workflow is properly conducted. To overcome this challenge, simple and straightforward identification of the source of origin of deer products is desired. For this reason, DNA barcoding technology is proposed to be an efficient method to perform species identification [16–18]. For animal identification, The mitochondrial cytochrome c oxidase subunit I (COI or cox1) gene sequence on the mitochondrial genome is generally considered to be the most useful DNA barcode, followed by other gene sequences such as cytochrome b (Cyt b) and ribosomal RNA 12S (rrn12). All of these loci are useful in distinguishing closely related animal species [19,20]. For TCM products, the DNA barcoding technique has been proven useful in the authentication of animal-based medicinal materials, including turtle shells [21], bear bile [22], and buffalo horns [23]. Although studies on the potential of DNA barcoding techniques for deer antlers, skin, and meat derived from *Cervus* have been carried out, in which they are restricted to the use of the cox1 sequences [24–27], no work has been conducted to evaluate the species discrimination strength of the DNA barcodes on other deer products, such as venison, bone, and fetus. To fill this research gap, we aimed to establish a simple and straightforward method to identify the source of origin of deer products using the DNA barcoding technique. A total of three different candidate DNA barcoding regions, including the cox1, Cyt b, and rrn12 genes, were evaluated for their species discrimination rates based on 22 deer product samples obtained from various sources. The findings of this study will provide an effective means for the trade management of medicinal materials as well as the food safety of deer products in the future.

## Materials and methods

### Sample collection

Sampling was carried out between July and August 2021. For the blind samples, a total of 22 deer products, of which 15 were thought to be derived from sika deer and seven from red deer, were purchased from various deer farms and pharmacies in Hainan Province. These blind samples were in the form of antler, bone, fetus, meat, penis, skin, and tail, in which their species of origin could not be identified through the naked eye. The species identity of each blind sample provided by the sellers was recorded, and they were not informed of the study beforehand. Two identified samples that are in the form of antlers were purchased from the China National Institute for Food and Drug Control (CNIFDC), of which SD-ref is derived from *C*. *nippon* (sika deer) and RD-ref is derived from *C*. *elaphus* (red deer) and were included as references. All the samples were oven-dried at low temperatures prior to being deposited at the Hainan Branch of the Institute of Medicinal Plant Development, Chinese Academy of Medical Sciences (Table 1).

**Table 1:** Information of the body part, form, place of purchase, DNA concentration and quality for the 22 blind samples and two reference samples used in this study.

Sample code	Label	Type	Purchase Place	DNA conc (ng/μL)	Purity 260/280
SD-ref	Sika deer antler	Medicinal powder	CNIFDC	677.5	1.89
RD-ref	Red deer antler	Medicinal powder	CNIFDC	623.2	1.90
SD01	Sika deer meat	Dry medicinal material	Medicinal materials market	996.4	1.97
SD02	Sika deer meat	Dry medicinal material	Medicinal materials market	969.2	1.98
SD03	Sika deer meat	Dry medicinal material	Medicinal materials market	856.4	1.97
SD04	Sika deer meat	Dry medicinal material	Medicinal materials market	839.6	1.97
SD05	Sika deer meat	Fresh sample	Deer farm	1041.6	1.97
SD06	Sika deer bone	Fresh sample	Deer farm	1246.4	1.92
SD07	Sika deer bone	Fresh sample	Deer farm	1215.0	1.91
SD08	Sika deer fetus	Dry medicinal material	Medicinal materials market	512.4	1.80
SD09	Sika deer fetus	Medicinal powder	Medicinal materials market	843.9	1.84
SD10	Sika deer antler	Dry medicinal material	Medicinal materials market	573.2	1.96
SD11	Sika deer antler	Fresh sample	Medicinal materials market	1272.1	1.89
SD12	Sika deer penis	Medicinal powder	Medicinal materials market	403.5	1.94
SD13	Sika deer tail	Dry medicinal material	Medicinal materials market	1081.8	2.00
SD14	Sika deer skin	Fresh sample	Deer farm	803.0	1.89
SD15	Sika deer wool	Fresh sample	Deer farm	106.9	1.99
RD01	Red deer meat	Dry medicinal material	Medicinal materials market	981.1	1.98
RD02	Red deer meat	Dry medicinal material	Medicinal materials market	911.6	1.96
RD03	Red deer meat	Dry medicinal material	Medicinal materials market	896.4	1.98
RD04	Red deer meat	Dry medicinal material	Medicinal materials market	888.6	1.99
RD05	Red deer meat	Fresh sample	Deer farm	1057.6	1.95
RD06	Red deer bone	Fresh sample	Deer farm	1133.2	1.90
RD07	Red deer bone	Fresh sample	Deer farm	1108.1	1.91

Note: SD-ref and RD-ref are reference samples; SD stands for Sika deer samples, and RD stands for Red deer samples.

### Total genomic DNA extraction

Before total genomic DNA extraction took place, the samples were surface sterilized with a 70% ethanol solution and cut into smaller sizes. With the help of liquid nitrogen, the samples were pulverized using a medium-flux tissue grinder (Dinghaoyuan Technology, China). Total genomic DNA extraction was carried out using an Omega Tissue DNA Kit D3396 (Omega Bio-technologies, USA) based on the manufacturer’s protocol with slight modifications. A total of 100 mg of pulverized sample material was added to a 1000 μL mixture of TL and BL buffers. The incubation time was increased to 6 h, while the subsequent steps followed the manufacturer’s protocol. The concentration and purity of the DNA extract were determined using Nanodrop 2000 (Thermo Scientific, USA).

### Polymerase chain reaction and sanger sequencing

Polymerase chain reaction (PCR) of the samples was carried out using three different primer sets that amplified three different gene regions, including cox1 (LCO1490: 5’-GGTCAACAAATCATAAAGATATTGG-3’, HCO2198: 5’-TAAACTTCAGGGTGACCAAAAAATCA-3’ [28]), Cyt b (L14724: 5’-GATATGAAAAACCATCGTTG-3’, H15149: 5’-CTCAGAATGATATTTGTCCTCA-3’ [29]), and rrn12 (L1091: 5’-AAACTGGGATTAGATACCCCACTAT-3’, H1478: 5’-GAGGGTGACGGGCGGTGTGT-3’ [30]). All reactions were carried out in a reaction mixture with a total final volume of 25 μL, containing 12.5 μL of 2×EcoTaq PCR Super Mix (Transgen Biotechnology, China), 1.0 μM of each primer, and 20 ng DNA template. PCR amplification was performed on an Easy Cycler 96C (Jena Analytical Instruments, Germany) with thermal settings programmed as follows: initial denaturation at 94°C for 3 min; 35 cycles of 94°C for 30 s, Ta for 30 s, and 72°C for 60 s; and a final extension at 72°C for 5 min. The Ta temperatures of the primer sets were 50°C, 54°C, and 54°C for cox1, Cyt b, and rrn12, respectively. Upon verification using a 1% agarose gel via electrophoresis and documentation under UV light, the PCR products were sent for Sanger sequencing from both ends at Ige Biotechnology Inc. (Guangdong, China). The results obtained from Sanger sequencing were aligned and edited for clean sequences using SeqMan, available in DNAStar v7.1 (Lasergene, Germany); the sequences from the reference species were deposited in the NCBI GenBank database for reference.

### Data analysis

A homology check was conducted using the NCBI BLAST program (http://blastncbi.nlm.nih.gov/Blast.cgi) to identify the reference species with similar sequence combinations to the blind samples. To provide a better resolution of the species identity of the blind samples, additional sequences of loci cox1, Cyt b, and rrn12 belonging to other closely related species of *C*. *elaphus* and *C*. *nippon* and four common adulterant species (known species with sequences over 400 bp for Cyt b and rrn12 and 600 bp for cox1) were mined from the GenBank database and were included in subsequent analysis. Atotal of 48 sequences were downloaded, which there were 16 each for cox1, Cyt b, and rrn12 (S1 Table). Multiple sequence alignment was conducted using the ClustalW program embedded in MEGA 11.0 [31]. Neighbor-joining (NJ) trees were generated based on the Kimura two-parameter (K2P) DNA substitution model under a complete deletion data treatment mode. The support values for each branch node were assessed with 1000 bootstrap replicates.

### Ethics statement

All materials used in the experiments were sourced from herbal trading markets and deer farms, and these collections were permitted and legal. Voucher specimens of all deer experimental materials were kept in the herbarium of the Analysis and Identification Center of the Hainan Branch of the Institute of Medicinal Plants, Chinese Academy of Medical Sciences.

## Results and discussion

### DNA analysis

The purity (ratio 260/280) of the total genomic DNA extracted from all 22 blind samples was in the acceptable range of 1.8 to 2.0, which indicated good DNA quality (Table 1). The DNA concentrations of the blind samples in the form of meat products, including RD01–05 and SD01–05, were between 800 and 1300 ng/μL; the bone products, including RD06, RD07, SD06, and SD07, were between 1100 and 1300 ng/μL, while other samples, including fetus, penis, antler, and wool, were less than 900 ng/μL. The blind sample that yielded the lowest DNA concentration was derived from a fresh wool specimen, which was 106.9 ng/μL. In addition, the blind samples that were fresh yielded higher DNA concentrations than those that were dried or processed into powder. This could be due to the degradation of DNA when drying or processing treatments were applied. In forensic identification, obtaining quality DNA that enables successful PCR amplification is considered the most crucial step in species identification. Note that DNA recovery in raw food always has a better yield than that in processed food [32]. Although successful PCR amplification can be achieved with only a minimum amount of DNA, e.g., 5 ng, which is not visible through the naked eye via gel electrophoresis, yielding sufficient DNA content for PCR amplification could be challenging when involving dry samples, such as hair, bone, and horn/antler [33], which is congruent with our findings. It is believed that the degradation rate of the DNA in dried samples is significant to the storage period; the longer the storage time is, the higher the degradation level. However, oral liquids and granules of Chinese patent medicine are mainly composed of aqueous extracts of TCM. In some cases, TCM pills, including deer product-derived medicines, and a certain amount of honey are usually added as an excipient. Such practices may hinder the DNA extraction and sequencing process [34]. Although an optimized DNA extraction protocol could improve the DNA yield from degraded samples, the improvised protocol used in this study exhibited satisfactory results–all blind samples were successfully amplified (100% success rate), suggesting that it should be useful for future applications.

### Sequence identity

Upon sequence assembly and after the primer adaptors were trimmed, the sequence lengths of the gene loci cox1, Cyt b, and rrn12 for the two reference samples as well as all the blind samples were 658, 424, and 394 bp, respectively (Table 2).

**Table 2:** Sequence length, and BLAST results for the three candidate DNA barcoding loci used in the study tested on the 22 blind samples and two reference samples.

Sample code	cox1 BLAST	Per Ident	Cyt b BLAST	Per Ident	rrn12 BLAST	Per Ident
SD-ref	*C*. *nippon* KX689229 (sika deer)	100%	*C*. *nippon* KR868807 (sika deer)	100%	*C*. *nippon* KX689229 (sika deer)	100%
RD-ref	*C*. *elaphus* OL679920 (red deer)	100%	*C*. *elaphus* OL679921 (red deer)	100%	*C*. *elaphus* OL679921 (red deer)	100%
SD01	*C*. *nippons* KX689229 (sika deer)	100%	*C*. *nippons* KR868807 (sika deer)	100%	*C*.*nippons* KX689229 (sika deer)	100%
SD02	*C*.*nippon* KX689229 (sika deer)	100%	*C*. *nippon* KR868807 (sika deer)	100%	*C*. *nippon* KX689229 (sika deer)	100%
SD03	*C*. *nippons* KX689229 (sika deer)	100%	*C*. *nippons* KR868807 (sika deer)	100%	*C*. *nippons* KX689229 (sika deer)	100%
SD04	*C*. *nippon* KX689229 (sika deer)	100%	*C*. *nippon* KR868807 (sika deer)	100%	*C*. *nippon* KX689229 (sika deer)	100%
SD05	*D*.*dama* KF509958 (fallow deer)	100%	*D*. *dama* NC_020700 (fallow deer)	100%	*D*. *dama* OQ473482 (fallow deer)	100%
SD06	*D*. *dama* KF509958 (fallow deer)	100%	*D*. *dama* NC_020700 (fallow deer)	100%	*D*. *dama* OQ473482 (fallow deer)	100%
SD07	*D*. *dama* KF509958 (fallow deer)	100%	*D*. *dama* NC_020700 (fallow deer)	100%	*D*. *dama* OQ473482 (fallow deer)	100%
SD08	*C*. *nippon* KX689229 (sika deer)	100%	*C*. *nippon* KR868807 (sika deer)	100%	*C*. *nippon* KX689229 (sika deer)	100%
SD09	*C*. *elaphus* OL679922 (red deer)	100%	*C*. *elaphus* OU343111 (red deer)	100%	*C*. *elaphus* KX868587 (red deer)	100%
SD10	*R*. *tarandus* MZ353653 (rein deer)	100%	*R*. *tarandus* MZ353653 (rein deer)	100%	*R*. *tarandus* MK608019 (rein deer)	100%
SD11	*C*. *elaphus* MW169471 (red deer)	100%	*C*. *elaphus* KM410147 (red deer)	100%	*C*. *elaphus* KX868587 (red deer)	100%
SD12	*C*. *elaphus* KX449334 (red deer)	100%	*C*. *elaphus* KX449334 (red deer)	100%	*C*. *elaphus* KX868587 (red deer)	100%
SD13	*C*. *nippon* KX689229 (sika deer)	100%	*C*. *nippon* KR868807 (sika deer)	100%	*C*. *nippon* KX689229 (sika deer)	100%
SD14	*C*. *nippon* KX689229 (sika deer)	100%	*C*. *nippon* KR868807 (sika deer)	100%	*C*. *nippon* KX689229 (sika deer)	100%
SD15	*C*. *nippon* KX689229 (sika deer)	100%	*C*. *nippon* KR868807 (sika deer)	100%	*C*. *nippon* KX689229 (sika deer)	100%
RD01	*C*. *elaphus* KX449334 (red deer)	100%	*C*. *elaphus* KF317925 (red deer)	100%	*C*. *elaphus* KX868587 (red deer)	100%
RD02	*C*. *elaphus* KX449334 (red deer)	100%	*C*. *elaphus* KF317925 (Red deer)	100%	*C*. *elaphus* KX868587 (red deer)	100%
RD03	*C*. *elaphus* KX449334 (red deer)	100%	*C*. *elaphus* KF317925 (red deer)	100%	*C*. *elaphus* KX868587 (red deer)	100%
RD04	*C*. *elaphus* KX449334 (red deer)	100%	*C*. *elaphus* KF317925 (red deer)	100%	*C*. *elaphus* KX868587 (red deer)	100%
RD05	*C*. *nippon* KX689229 (sika deer)	100%	*C*. *nippon* KR868807 (sika deer)	100%	*C*. *nippon* KX689229 (sika deer)	100%
RD06	*C*. *nippon* KX689229 (sika deer)	100%	*C*. *nippon* KR868807 (sika deer)	100%	*C*. *nippon* KX689229 (sika deer)	100%
RD07	*C*. *nippon* KX689229 (sika deer)	100%	*C*. *nippon* KR868807 (sika deer)	100%	*C*. *nippon* KX689229 (sika deer)	100%

All the blind samples that were subjected to BLAST analysis returned a match with the record deposited in NCBI GenBank (Table 2). A powerful DNA barcoding locus contains sufficient single nucleotide polymorphisms to tell close species apart [35], while BLAST analysis relies on the sequence similarity of the query sequence to the reference sequence deposited in the GenBank database [36]. The sequence alignments for cox1, Cyt b, and rrn12 of the two reference samples resulted in 20, 21, and eight variable sites, respectively (Table 3). With the assumption that the species identities of the reference sequences deposited in the GenBank database are deemed accurate, species identification can be regarded as correctly assigned when all three candidate DNA barcoding loci indicate the same species under a match identity of 100%. Based on such criteria, all the blind samples were indicated with the same species identity when using the three candidate DNA barcoding loci. All 22 blind samples revealed the same species identity through BLAST analysis using the three candidate DNA barcoding loci used, with at least 100% match identity.

**Table 3:** Nucleotide variation in the cox1, Cyt b and rrn12 region of SD-ref compared to RD-ref.

Items	Nucleotide positions and variation																				
cox1	14	43	92	94	103	106	166	178	217	235	289	290	367	386	409	415	505	571	596	610	
*C*. *nippon*	C	A	C	G	T	T	A	C	T	C	T	T	C	C	G	T	C	C	C	T	
*C*. *elaphus*	T	G	T	A	C	C	G	T	C	T	C	C	T	T	A	C	T	T	T	C	
Cyt b	49	50	76	88	121	127	139	143	157	178	196	211	250	265	301	319	358	361	376	379	412
*C*. *nippon*	A	T	C	C	T	C	T	C	T	A	C	T	T	C	C	A	A	G	C	C	A
*C*. *elaphus*	G	C	T	A	C	A	C	T	C	G	T	C	C	T	T	G	G	A	T	T	G
rrn12	28	96	160	188	233	304	323	377													
*C*. *nippon*	T	T	C	A	A	C	C	T													
*C*. *elaphus*	C	C	A	G	G	T	T	C													

Based on the BLAST results, we managed to group the 22 blind samples into four different species identities: *C*. *elaphus*, *C*. *nippon*, *D*. *dama*, and *R*. *tarandus*. Among the 22 blind samples tested in this study, 11 (RD05-07, SD01-04, SD08, SD13-15) were identified as *C*. *nippon*; seven (RD01-04, SD09, SD11-12) were identified as *C*. *elaphus*; three (SD05-07) were identified as *D*. *dama*; and one (SD10) was identified as *R*. *tarandus*. Incongruence between the species identity that was provided by the seller and the result from the BLAST analysis was detected among the ten blind samples tested. Based on the BLAST results, three of the blind samples (SD09, SD11-12) that were derived from red deer (*C*. *elaphus*) were misidentified by the seller to originate from sika deer (*C*. *nippon*), while four samples (SD05-07, SD10) were adulterated with body parts from fallow deer (*D*. *dama*) and reindeer (*R*. *tarandus*). And three blind samples (RD01-07) that were thought to originate from red deer (*C*. *elaphus*), were identified as sika deer (*C*. *nippon*).

**Fig 1:**
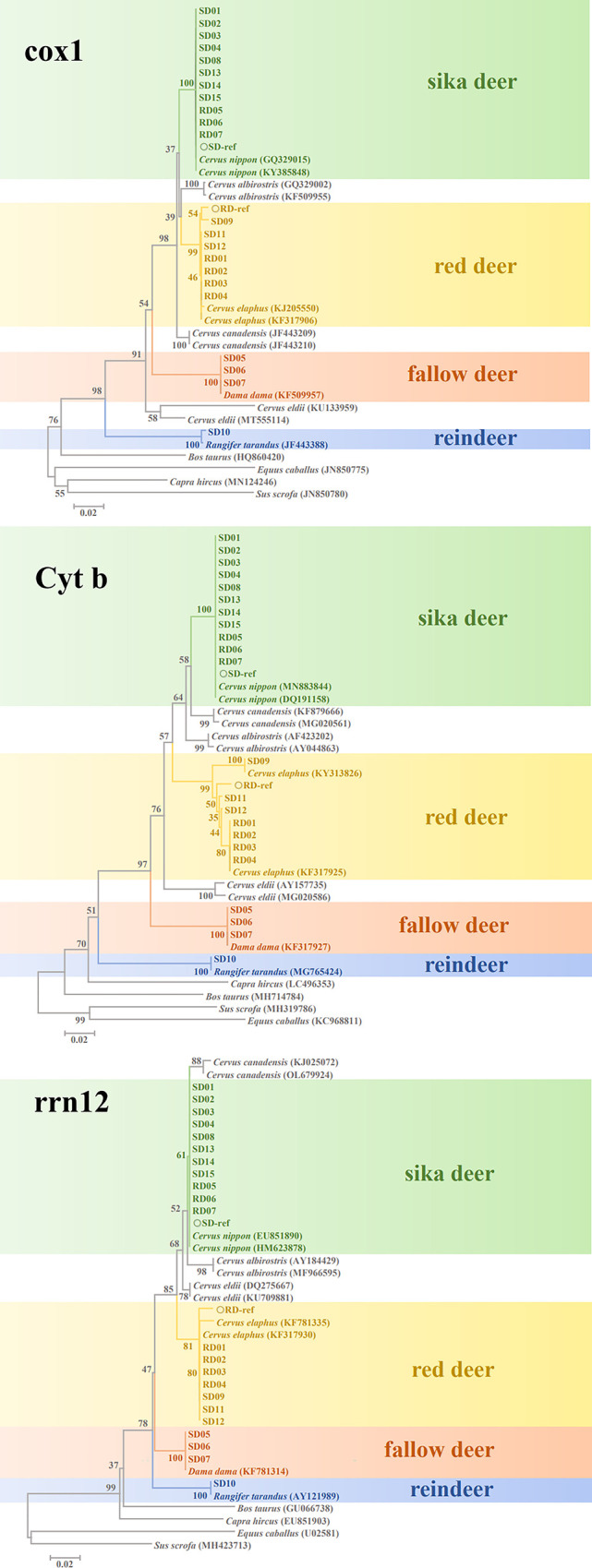
Phylogenetic tree of cox1, Cyt b and rrn12 gene sequences.

The NJ trees based on cox1, Cyt b, and rrn12 analyzed separately revealed that all species were clustered in their respective branches. For DNA barcoding analysis, species delineation is considered to be established based on a selected DNA barcoding locus when all species are clustered under different branches, while individuals of the same species are clustered under the same branch; the genetic distance at the intraspecific level should not exceed that at the interspecific level [16]. Thus, it is assured that all three proposed DNA barcoding loci are potentially useful in distinguishing *C*. *elaphus* and *C*. *nippon* from their adulterants using the DNA barcoding technique. Following the clustering pattern of each NJ tree, it can be concluded that seven blind samples (RD01-04, SD09, SD11-12) were derived from *C*. *elaphus*; 11 blind samples (RD05-07, SD01-04, SD08, SD13-15) were derived from *C*. *nippon*; three blind samples (SD05-07) were derived from *D*. *dama*; and one blind sample (SD10) was derived from *R*. *tarandus*. The results from the NJ tree are congruent with those from the BLAST analysis. Based on the three NJ trees, we proposed that the candidate DNA barcoding loci cox1 and Cyt b are suitable for the identification and authentication of deer products derived from *C*. *elaphus* and *C*. *nippon*. Although weak bootstrap support values are detected in the NJ trees, the branch support value is not considered an important feature in DNA barcoding analysis [16]. The candidate DNA barcoding locus, rrn12, was not selected as a potential DNA barcode in this study because the sequence variation between *C*. *nippon* and *C*. *canadensis* was not sufficient to separate the branches of the two species distinctly (Fig 1), making it less advantageous than the other two candidate DNA barcoding loci. An ideal genetic variation in barcoding should be significantly greater between species than within species and form a distinct interval between them, called the barcoding gap [37]. As long as the barcoding gap of different species is higher than that of the same species, it is generally considered that this fragment can be used for DNA barcoding identification. It is noteworthy to mention that the standards obtained from the CNIFDC were correctly identified at the species level; RD-ref and SD-ref were derived from *C*. *elaphus* and *C*. *nippon*, respectively. To provide a reference for accurate species identification, the sequences for both reference samples were deposited into NCBI GenBank under the accession numbers OP849627 and OP849628 (cox1), OP866991 and OP866992 (Cyt b), and OP856556 and OP856557 (rrn12).

Adulteration of food and TCM material has become a growing concern. In recent years, the adulteration of TCM has affected people’s health and drug efficacy [38]. Deer products from sika deer and red deer are considered authentic in China; however, due to the lack of effective identification means, dishonest traders not only might replace deer products with body parts from adulterant species, but there are cases where authentic deer products were replaced with body parts from other deer species aside from *C*. *elaphus* and *C*. *nippon*, such as fallow deer, mule deer, reindeer, sambar, etc. [39], as demonstrated in this study for blind samples SD05-07 and SD10. As SD05-07 were purchased from a deer farm, it is possible that the traders or farmers are trying to take advantage of their clients by convincing them that they are actually selling only deer products from *C*. *elaphus* and/or *C*. *nippon* from their farm.

Species differentiation between *D*. *dama* and the two *Cervus* species has been proposed to be carried out using skeletal or teeth features [40,41] but not meat. At the molecular level, studies to distinguish these taxa are limited to real-time PCR [42] and PCR-RFLP [43] techniques. For real-time PCR, the technique is suitable to detect the presence of DNA from different closely related species in processed/unprocessed meat mixtures [44]; however, a set of reference DNA is required every time the experiment is conducted. Unlike real-time PCR, DNA barcoding utilizes the reference sequences that are deposited in NCBI GenBank for comparison and phylogenetic tree reconstruction. Furthermore, experiments based on the real-time PCR technique are not cost-effective when compared to the DNA barcoding technique [45]; Taq polymerase, fluorescent dye, and qPCR mix are relatively more expensive when compared to conventional PCR mix. For PCR-RFLP, there is a possibility of inconsistency in the results, i.e., differences in banding patterns for the same species due to genetic variation [46]. However, the technique is labor intensive and time-consuming, and preknowledge of a suitable endonuclease that could be used for digestion is required to ensure that differences in banding patterns between closely related species can be achieved prior to the experiment [47]. A nondestructive method using X-ray microtomography coupled with an SVM classifier was proposed to be useful in differentiating the species of origin for reindeer and red deer antlers [48], in which species differentiation can be carried out by analyzing the alveoli on their antlers. However, such an identification method is known to be useful for prehistoric studies [49], but not in food control due to the need for sophisticated machines and data analyses. Although the consumption of body parts derived from both adulterant species, *D*. *dama* and *R*. *tarandus*, would not result in any hazardous effects to human health, we speculated that the traders were either confused and misidentified the products beforehand, or the deer products were purposely replaced with those from an adulterant species as a fraudulent and business profit sham. Nevertheless, without a proper farming or handling system, adulterated products cannot be assumed to be safe and free of toxic ingredients at all times; in addition, a decline in medicinal quality is expected [50]. Therefore, effective species authentication methods are deemed requisite to ensure the safety and efficacy of TCM, which indirectly provides great significance to the internationalization of the TCM industry.

## Conclusion

In this study, the DNA extraction method used is suitable for various forms of deer products, including meat, bones, antlers, etc., some of which were processed into dry or powder form. By using the extracted DNA for subsequent experiments, species identification and authentication of deer products were carried out via BLAST and DNA barcoding analyses. Among the three candidate DNA barcoding loci tested in this study, the gene sequences for cox1 and Cyt b are proposed to be useful for the DNA barcoding of deer products derived from *C*. *elaphus* and *C*. *nippon*, while the third gene locus, rrn12, exhibited a less advantageous discrimination strength in the DNA barcoding analysis when compared to the other two gene loci. As a result, three blind samples that were labeled as originating from red deer were actually derived from *C*. *nippon*, while the presence of adulterant species derived from *D*. *dama* and *R*. *tarandus* was detected in four blind samples that were labeled as originating from sika deer. Based on the findings of this study, the DNA barcoding technique has been proven to be applicable in species identification and authentication of deer products and could therefore effectively prevent deer product trade fraud and ensure the safe use of authentic deer products.

## Supporting information

S1 Table GenBank accession numbers for the reference sequences used in the study. (DOCX)

S1 File Sequencing results of 24 samples of cox1, cytb, and rrn12 in this study. (DOCX)
