# Supplementary material for: DNA barcoding for the identification and authentication of medicinal deer (Cervus sp.) products in China
Source: PLoS One. 2024 Jan 19;19(1):e0297164. doi: 10.1371/journal.pone.0297164 (PMC10798443; doi:10.1371/journal.pone.0297164)
Supplement: S1 Table — (DOCX) [file pone.0297164.s001.docx]

**Supplementary information**

**S1 Table. GenBank accession numbers for the reference sequences used in the study**

| Family | Species | cox1 | Cyt b | rrn12 |
| --- | --- | --- | --- | --- |
| Cervidae | *Cervus albirostris* | GQ329002, KF509955 | AF423202, AY044863 | AY184429, MF966595 |
|  | *Cervus canadensis* | JF443209, JF443210 | KF879666, MG020561 | KJ025072, OL679924 |
|  | *Cervus elaphus* | KF317906, KJ205550 | AB924664, KF133899 | KF781335, KF317930 |
|  | *Cervus eldii* | KU133959, MT555114 | AY157735, MG020586 | DQ275667, KU709881 |
|  | *Cervus nippon* | KY385848, GQ329015 | MN883844, DQ191158 | EU851890, HM623878 |
|  | *Dama dama* | KF509957 | AJ000022 | KF781314 |
|  | *Rangifertarandus* | KX05248 | DQ673134 | AY121989 |
| Bovidae | *Bos taurus* | HQ860420 | MH714784 | GU066738 |
|  | *Capra hircus* | MN124246 | KT283248 | EU851903 |
| Equidae | *Equus caballus* | JN850775 | KC968811 | U02581 |
| Suidae | *Sus scrofa* | JN850780 | MH319786 | MH423713 |
