# Supplementary material for: DNA barcoding for the identification and authentication of medicinal deer (Cervus sp.) products in China
Source: PLoS One. 2024 Jan 19;19(1):e0297164. doi: 10.1371/journal.pone.0297164 (PMC10798443; doi:10.1371/journal.pone.0297164)
Supplement: S1 File — (DOCX) [file pone.0297164.s002.docx]

**Supplementary information**

**S1file. Sequencing results and Genbank accession numbers of 22 samples of cox1, cytb, and rrn12 in this study.**

1. **Raw sequence of cox1**

SD-ref (accession number OP849627)

TACTCTGTATCTACTATTTGGTGCCTGAGCAGGCATAGTAGGAACAGCCTTAAGCCTACTGATTCGTGCCGAACTGGGCCAACCTGGTACTCTGCTTGGAGATGATCAAATTTATAATGTTATCGTAACCGCACATGCATTCGTAATAATTTTCTTTATAGTTATACCAATTATAATCGGAGGATTTGGTAATTGACTAGTTCCCCTAATAATTGGTGCCCCAGACATAGCATTCCCTCGAATAAACAATATAAGCTTTTGACTCCTCCCTCCTTCTTTCTTACTACTTTTAGCATCATCTATAGTTGAAGCTGGCGCAGGAACAGGCTGAACTGTATATCCCCCTCTAGCTGGCAACTTAGCTCACGCAGGGGCTTCAGTAGACCTGACCATTTTTTCTTTACACTTGGCAGGTGTCTCCTCAATTCTAGGGGCCATTAACTTTATTACAACAATTATCAATATAAAACCCCCTGCCATATCACAATATCAAACCCCTCTATTCGTGTGATCCGTATTAGTCACTGCTGTACTACTACTTCTCTCACTCCCTGTACTAGCAGCCGGAATCACAATACTATTAACAGACCGAAACCTAAATACAACCTTTTTTGACCCAGCAGGAGGCGGAGATCCTATTCTATATCAACACTTGTTC

RD-ref (accession number OP849628)

TACTCTGTATCTATTATTTGGTGCCTGAGCAGGCATAGTAGGGACAGCCTTAAGCCTACTGATTCGTGCCGAACTGGGCCAACCTGGTACTTTACTTGGAGACGACCAAATTTATAATGTTATCGTAACCGCACATGCATTCGTAATAATTTTCTTTATAGTTATGCCAATTATAATTGGAGGATTTGGTAATTGACTAGTTCCCCTAATAATTGGCGCCCCAGACATAGCATTTCCTCGAATAAACAATATAAGCTTTTGACTCCTCCCTCCTTCTTTCTTACTACTCCTAGCATCATCTATAGTTGAAGCTGGCGCAGGAACAGGCTGAACTGTATATCCCCCTCTAGCTGGCAACTTAGCTCATGCAGGGGCTTCAGTAGACTTGACCATTTTTTCTTTACACTTAGCAGGCGTCTCCTCAATTCTAGGGGCCATTAACTTTATTACAACAATTATCAATATAAAACCCCCTGCCATATCACAATATCAAACCCCTCTATTTGTGTGATCCGTATTAGTCACTGCTGTACTACTACTTCTCTCACTCCCTGTACTAGCAGCCGGAATTACAATACTATTAACAGACCGAAACTTAAATACAACCTTCTTTGACCCAGCAGGAGGCGGAGATCCTATTCTATATCAACACTTGTTC

SD01

TACTCTGTATCTACTATTTGGTGCCTGAGCAGGCATAGTAGGAACAGCCTTAAGCCTACTGATTCGTGCCGAACTGGGCCAACCTGGTACTCTGCTTGGAGATGATCAAATTTATAATGTTATCGTAACCGCACATGCATTCGTAATAATTTTCTTTATAGTTATACCAATTATAATCGGAGGATTTGGTAATTGACTAGTTCCCCTAATAATTGGTGCCCCAGACATAGCATTCCCTCGAATAAACAATATAAGCTTTTGACTCCTCCCTCCTTCTTTCTTACTACTTTTAGCATCATCTATAGTTGAAGCTGGCGCAGGAACAGGCTGAACTGTATATCCCCCTCTAGCTGGCAACTTAGCTCACGCAGGGGCTTCAGTAGACCTGACCATTTTTTCTTTACACTTGGCAGGTGTCTCCTCAATTCTAGGGGCCATTAACTTTATTACAACAATTATCAATATAAAACCCCCTGCCATATCACAATATCAAACCCCTCTATTCGTGTGATCCGTATTAGTCACTGCTGTACTACTACTTCTCTCACTCCCTGTACTAGCAGCCGGAATCACAATACTATTAACAGACCGAAACCTAAATACAACCTTTTTTGACCCAGCAGGAGGCGGAGATCCTATTCTATATCAACACTTGTTC

SD02

TACTCTGTATCTACTATTTGGTGCCTGAGCAGGCATAGTAGGAACAGCCTTAAGCCTACTGATTCGTGCCGAACTGGGCCAACCTGGTACTCTGCTTGGAGATGATCAAATTTATAATGTTATCGTAACCGCACATGCATTCGTAATAATTTTCTTTATAGTTATACCAATTATAATCGGAGGATTTGGTAATTGACTAGTTCCCCTAATAATTGGTGCCCCAGACATAGCATTCCCTCGAATAAACAATATAAGCTTTTGACTCCTCCCTCCTTCTTTCTTACTACTTTTAGCATCATCTATAGTTGAAGCTGGCGCAGGAACAGGCTGAACTGTATATCCCCCTCTAGCTGGCAACTTAGCTCACGCAGGGGCTTCAGTAGACCTGACCATTTTTTCTTTACACTTGGCAGGTGTCTCCTCAATTCTAGGGGCCATTAACTTTATTACAACAATTATCAATATAAAACCCCCTGCCATATCACAATATCAAACCCCTCTATTCGTGTGATCCGTATTAGTCACTGCTGTACTACTACTTCTCTCACTCCCTGTACTAGCAGCCGGAATCACAATACTATTAACAGACCGAAACCTAAATACAACCTTTTTTGACCCAGCAGGAGGCGGAGATCCTATTCTATATCAACACTTGTTC

SD03

TACTCTGTATCTACTATTTGGTGCCTGAGCAGGCATAGTAGGAACAGCCTTAAGCCTACTGATTCGTGCCGAACTGGGCCAACCTGGTACTCTGCTTGGAGATGATCAAATTTATAATGTTATCGTAACCGCACATGCATTCGTAATAATTTTCTTTATAGTTATACCAATTATAATCGGAGGATTTGGTAATTGACTAGTTCCCCTAATAATTGGTGCCCCAGACATAGCATTCCCTCGAATAAACAATATAAGCTTTTGACTCCTCCCTCCTTCTTTCTTACTACTTTTAGCATCATCTATAGTTGAAGCTGGCGCAGGAACAGGCTGAACTGTATATCCCCCTCTAGCTGGCAACTTAGCTCACGCAGGGGCTTCAGTAGACCTGACCATTTTTTCTTTACACTTGGCAGGTGTCTCCTCAATTCTAGGGGCCATTAACTTTATTACAACAATTATCAATATAAAACCCCCTGCCATATCACAATATCAAACCCCTCTATTCGTGTGATCCGTATTAGTCACTGCTGTACTACTACTTCTCTCACTCCCTGTACTAGCAGCCGGAATCACAATACTATTAACAGACCGAAACCTAAATACAACCTTTTTTGACCCAGCAGGAGGCGGAGATCCTATTCTATATCAACACTTGTTC

SD04

TACTCTGTATCTACTATTTGGTGCCTGAGCAGGCATAGTAGGAACAGCCTTAAGCCTACTGATTCGTGCCGAACTGGGCCAACCTGGTACTCTGCTTGGAGATGATCAAATTTATAATGTTATCGTAACCGCACATGCATTCGTAATAATTTTCTTTATAGTTATACCAATTATAATCGGAGGATTTGGTAATTGACTAGTTCCCCTAATAATTGGTGCCCCAGACATAGCATTCCCTCGAATAAACAATATAAGCTTTTGACTCCTCCCTCCTTCTTTCTTACTACTTTTAGCATCATCTATAGTTGAAGCTGGCGCAGGAACAGGCTGAACTGTATATCCCCCTCTAGCTGGCAACTTAGCTCACGCAGGGGCTTCAGTAGACCTGACCATTTTTTCTTTACACTTGGCAGGTGTCTCCTCAATTCTAGGGGCCATTAACTTTATTACAACAATTATCAATATAAAACCCCCTGCCATATCACAATATCAAACCCCTCTATTCGTGTGATCCGTATTAGTCACTGCTGTACTACTACTTCTCTCACTCCCTGTACTAGCAGCCGGAATCACAATACTATTAACAGACCGAAACCTAAATACAACCTTTTTTGACCCAGCAGGAGGCGGAGATCCTATTCTATATCAACACTTGTTC

SD05

TACCCTGTATCTACTATTTGGTGcCTGAGCAGGCATAGTAGGAACAGCTTTAAGCCTATTGATTCGTGCTGAACTGGGCCAACCTGGTACCCTACTTGGAGATGACCAAATTTATAATGTTATTGTAACCGCACATGCATTCGTAATAATTTTCTTTATAGTTATACCAATTATAATCGGAGGATTTGGTAACTGACTAGTTCCCTTAATAATTGGTGCCCCAGATATAGCATTCCCTCGAATAAACAATATGAGCTTTTGACTCCTTCCTCCCTCTTTCTTACTACTTCTAGCATCATCTATAGTTGAAGCTGGCGCAGGAACAGGCTGAACTGTGTACCCCCCTCTAGCTGGTAACTTAGCTCACGCAGGAGCCTCAGTGGACCTAACTATCTTTTCTCTACACCTGGCAGGTGTCTCTTCAATTCTAGGGGCCATTAACTTTATTACAACAATTATCAATATAAAACCCCCTGCTATGTCACAATACCAAACTCCCCTATTTGTGTGATCCGTACTAGTCACTGCTGTATTACTACTTCTCTCACTCCCAGTACTAGCAGCTGGAATTACAATATTATTAACAGACCGAAATTTAAATACAACCTTTTTTGATCCAGCAGGAGGCGGAGATCCCATTCTATATCAACACTTATTC

SD06

TACCCTGTATCTACTATTTGGTGCCTGAGCAGGCATAGTAGGAACAGCTTTAAGCCTATTGATTCGTGCTGAACTGGGCCAACCTGGTACCCTACTTGGAGATGACCAAATTTATAATGTTATTGTAACCGCACATGCATTCGTAATAATTTTCTTTATAGTTATACCAATTATAATCGGAGGATTTGGTAACTGACTAGTTCCCTTAATAATTGGTGCCCCAGATATAGCATTCCCTCGAATAAACAATATGAGCTTTTGACTCCTTCCTCCCTCTTTCTTACTACTTCTAGCATCATCTATAGTTGAAGCTGGCGCAGGAACAGGCTGAACTGTGTACCCCCCTCTAGCTGGTAACTTAGCTCACGCAGGAGCCTCAGTGGACCTAACTATCTTTTCTCTACACCTGGCAGGTGTCTCTTCAATTCTAGGGGCCATTAACTTTATTACAACAATTATCAATATAAAACCCCCTGCTATGTCACAATACCAAACTCCCCTATTTGTGTGATCCGTACTAGTCACTGCTGTATTACTACTTCTCTCACTCCCAGTACTAGCAGCTGGAATTACAATATTATTAACAGACCGAAATTTAAATACAACCTTTTTTGATCCAGCAGGAGGCGGAGATCCCATTCTATATCAACACTTATTC

SD07

TACCCTGTATCTACTATTTGGTGCCTGAGCAGGCATAGTAGGAACAGCTTTAAGCCTATTGATTCGTGCTGAACTGGGCCAACCTGGTACCCTACTTGGAGATGACCAAATTTATAATGTTATTGTAACCGCACATGCATTCGTAATAATTTTCTTTATAGTTATACCAATTATAATCGGAGGATTTGGTAACTGACTAGTTCCCTTAATAATTGGTGCCCCAGATATAGCATTCCCTCGAATAAACAATATGAGCTTTTGACTCCTTCCTCCCTCTTTCTTACTACTTCTAGCATCATCTATAGTTGAAGCTGGCGCAGGAACAGGCTGAACTGTGTACCCCCCTCTAGCTGGTAACTTAGCTCACGCAGGAGCCTCAGTGGACCTAACTATCTTTTCTCTACACCTGGCAGGTGTCTCTTCAATTCTAGGGGCCATTAACTTTATTACAACAATTATCAATATAAAACCCCCTGCTATGTCACAATACCAAACTCCCCTATTTGTGTGATCCGTACTAGTCACTGCTGTATTACTACTTCTCTCACTCCCAGTACTAGCAGCTGGAATTACAATATTATTAACAGACCGAAATTTAAATACAACCTTTTTTGATCCAGCAGGAGGCGGAGATCCCATTCTATATCAACACTTATTC

SD08

TACTCTGTATCTACTATTTGGTGCCTGAGCAGGCATAGTAGGAACAGCCTTAAGCCTACTGATTCGTGCCGAACTGGGCCAACCTGGTACTCTGCTTGGAGATGATCAAATTTATAATGTTATCGTAACCGCACATGCATTCGTAATAATTTTCTTTATAGTTATACCAATTATAATCGGAGGATTTGGTAATTGACTAGTTCCCCTAATAATTGGTGCCCCAGACATAGCATTCCCTCGAATAAACAATATAAGCTTTTGACTCCTCCCTCCTTCTTTCTTACTACTTTTAGCATCATCTATAGTTGAAGCTGGCGCAGGAACAGGCTGAACTGTATATCCCCCTCTAGCTGGCAACTTAGCTCACGCAGGGGCTTCAGTAGACCTGACCATTTTTTCTTTACACTTGGCAGGTGTCTCCTCAATTCTAGGGGCCATTAACTTTATTACAACAATTATCAATATAAAACCCCCTGCCATATCACAATATCAAACCCCTCTATTCGTGTGATCCGTATTAGTCACTGCTGTACTACTACTTCTCTCACTCCCTGTACTAGCAGCCGGAATCACAATACTATTAACAGACCGAAACCTAAATACAACCTTTTTTGACCCAGCAGGAGGCGGAGATCCTATTCTATATCAACACTTGTTC

SD09

TACTCTGTATCTATTATTTGGTGCCTGAGCAGGCATAGTAGGGACGGCCTTAAGCCTACTGATTCGTGCCGAACTGGGCCAACCTGGTACTCTACTTGGAGATGACCAAATTTATAATGTTATCGTAACCGCACATGCATTCGTAATAATTTTCTTTATAGTTATGCCAATTATAATTGGAGGATTTGGTAATTGACTAGTTCCCCTAATAATTGGCGCCCCAGACATAGCATTTCCTCGAATAAACAATATAAGCTTTTGACTCCTCCCTCCTTCTTTCTTACTACTCCTAGCATCATCTATAGTTGAAGCTGGCGCAGGAACAGGCTGAACTGTATATCCCCCTCTAGCTGGCAACTTAGCTCATGCAGGGGCTTCAGTAGACTTGACTATTTTTTCTTTACACTTAGCAGGCGTCTCCTCAATTCTGGGGGCCATTAACTTTATTACAACAATTATCAATATAAAACCCCCTGCCATATCACAATATCAAACCCCTCTATTTGTGTGATCCGTATTAGTCACTGCTGTACTACTACTTCTCTCACTCCCTGTACTAGCAGCCGGAATTACAATACTATTAACAGACCGAAACTTAAATACAACCTTCTTTGACCCAGCAGGAGGCGGAGATCCTATTCTATATCAACACTTATTC

SD10

CACCTTGTATTTACTATTTGGTGCTTGAGCAGGCATAGTAGGAACTGCCCTAAGCTTACTAATCCGTGCTGAACTGGGCCAACCTGGGACCCTACTCGGAGACGATCAAATTTATAATGTAATTGTAACCGCACATGCATTCGTAATAATTTTCTTTATAGTAATGCCAATTATAATTGGAGGATTTGGTAATTGACTTGTCCCTCTAATAATTGGTGCCCCAGATATAGCATTCCCTCGGATAAATAATATAAGCTTCTGACTTCTCCCTCCCTCTTTTCTACTTCTTCTAGCATCATCCATAATTGAAGCTGGGGCAGGAACAGGTTGAACTGTTTACCCTCCTTTAGCTGGTAACCTAGCTCACGCAGGAGCTTCAGTAGACTTAACTATTTTCTCTTTACACTTAGCAGGTGTCTCCTCAATTTTAGGAGCAATTAACTTTATCACAACAATTATTAATATAAAACCTCCTGCTATATCACAGTATCAAACCCCTTTATTTGTATGATCTGTCTTAATCACTGCTGTATTATTACTTCTCTCACTTCCTGTACTAGCAGCCGGAATTACAATACTACTAACAGACCGAAATTTAAATACAACTTTCTTCGACCCAGCAGGAGGCGGGGATCCCATCCTATATCAACATTTATTC

SD11

TACTCTGTATATATTATTTGGTGCCTGAGCAGGCATAGTAGGGACAGCCTTAAGCCTACTGATTCGTGCCGAACTGGGCCAACCTGGTACTCTACTTGGAGATGACCAAATTTATAATGTTATCGTAACCGCRCATGCATTCGTAATAATTTTCTTTATAGTTATGCCAATTATAATTGGAGGATTTGGTAATTGACTAGTTCCCCTAATAATTGGCGCCCCAGACATAGCATTTCCTCGAATAAACAATATAAGCTTTTGACTCCTCCCTCCTTCTTTCTTACTACTCCTAGCATCATCTATAGTTGAAGCTGGCGCAGGAACAGGCTGAACTGTATATCCCCCTCTAGCTGGCAACTTAGCTCATGCAGGGGCTTCAGTAGACCTGACTATTTTTTCTTTACACTTAGCAGGCGTCTCCTCAATTCTAGGGGCCATTAACTTTATTACAACAATTATCAATATAAAACCCCCTGCCATATCACAATATCAAACCCCTCTATTTGTGTGATCCGTATTAGTCACTGCTGTACTACTACTTCTCTCACTCCCTGTACTAGCAGCCGGAATTACAATACTATTAACAGACCGAAACTTAAATACAACCTTCTTTGACCCAGCAGGAGGCGGAGATCCTATTCTATATCAACACTTATTC

SD12

TACTCTGTATCTATTATTTGGTGCCTGAGCAGGCATAGTAGGGACAGCCTTAAGCCTACTGATTCGTGCCGAACTGGGCCAACCTGGTACTCTWCTTGGAGATGACCAAATTTATAATGTTATCGTAACCGCACATGCATTCGTAATAATTTTCTTTATAGTTATGCCAATTATAATTGGAGGATTTGGTAATTGACTAGTTCCCCTAATAATTGGCGCCCCAGACATAGCATTTCCTCGAATAAACAATATAAGCTTTTGACTCCTCCCTCCTTCTTTCTTACTACTCCTAGCATCATCTATAGTTGAAGCTGGCGCAGGAACAGGCTGAACTGTATATCCCCCTCTAGCTGGCAACTTAGCTCATGCAGGGGCTTCAGTAGACCTGACTATTTTTTCTTTACACTTAGCAGGCGTCTCCTCAATTCTAGGGGCCATTAACTTTATTACAACAATTATCAATATAAAACCCCCTGCCATATCACAATATCAAACCCCTCTATTTGTGTGATCCGTATTAGTCACTGCTGTACTACTACTTCTCTCACTCCCTGTACTAGCAGCCGGAATTACAATACTATTAACAGACCGAAACTTAAATACAACCTTCTTTGACCCAGCAGGAGGCGGAGATCCTATtCTATATCAACACTTGTTC

SD13

TACTCTGTATCTACTATTTGGTGCCTGAGCAGGCATAGTAGGAACAGCCTTAAGCCTACTGATTCGTGCCGAACTGGGCCAACCTGGTACTCTGCTTGGAGATGATCAAATTTATAATGTTATCGTAACCGCACATGCATTCGTAATAATTTTCTTTATAGTTATACCAATTATAATCGGAGGATTTGGTAATTGACTAGTTCCCCTAATAATTGGTGCCCCAGACATAGCATTCCCTCGAATAAACAATATAAGCTTTTGACTCCTCCCTCCTTCTTTCTTACTACTTTTAGCATCATCTATAGTTGAAGCTGGCGCAGGAACAGGCTGAACTGTATATCCCCCTCTAGCTGGCAACTTAGCTCACGCAGGGGCTTCAGTAGACCTGACCATTTTTTCTTTACACTTGGCAGGTGTCTCCTCAATTCTAGGGGCCATTAACTTTATTACAACAATTATCAATATAAAACCCCCTGCCATATCACAATATCAAACCCCTCTATTCGTGTGATCCGTATTAGTCACTGCTGTACTACTACTTCTCTCACTCCCTGTACTAGCAGCCGGAATCACAATACTATTAACAGACCGAAACCTAAATACAACCTTTTTTGACCCAGCAGGAGGCGGAGATCCTATTCTATATCAACACTTGTTC

SD14

TACTCTGTATCTACTATTTGGTGCCTGAGCAGGCATAGTAGGAACAGCCTTAAGCCTACTGATTCGTGCCGAACTGGGCCAACCTGGTACTCTGCTTGGAGATGATCAAATTTATAATGTTATCGTAACCGCACATGCATTCGTAATAATTTTCTTTATAGTTATACCAATTATAATCGGAGGATTTGGTAATTGACTAGTTCCCCTAATAATTGGTGCCCCAGACATAGCATTCCCTCGAATAAACAATATAAGCTTTTGACTCCTCCCTCCTTCTTTCTTACTACTTTTAGCATCATCTATAGTTGAAGCTGGCGCAGGAACAGGCTGAACTGTATATCCCCCTCTAGCTGGCAACTTAGCTCACGCAGGGGCTTCAGTAGACCTGACCATTTTTTCTTTACACTTGGCAGGTGTCTCCTCAATTCTAGGGGCCATTAACTTTATTACAACAATTATCAATATAAAACCCCCTGCCATATCACAATATCAAACCCCTCTATTCGTGTGATCCGTATTAGTCACTGCTGTACTACTACTTCTCTCACTCCCTGTACTAGCAGCCGGAATCACAATACTATTAACAGACCGAAACCTAAATACAACCTTTTTTGACCCAGCAGGAGGCGGAGATCCTATTCTATATCAACACTTGTTC

SD15

TACTCTGTATCTACTATTTGGTGCCTGAGCAGGCATAGTAGGAACAGCCTTAAGCCTACTGATTCGTGCCGAACTGGGCCAACCTGGTACTCTGCTTGGAGATGATCAAATTTATAATGTTATCGTAACCGCACATGCATTCGTAATAATTTTCTTTATAGTTATACCAATTATAATCGGAGGATTTGGTAATTGACTAGTTCCCCTAATAATTGGTGCCCCAGACATAGCATTCCCTCGAATAAACAATATAAGCTTTTGACTCCTCCCTCCTTCTTTCTTACTACTTTTAGCATCATCTATAGTTGAAGCTGGCGCAGGAACAGGCTGAACTGTATATCCCCCTCTAGCTGGCAACTTAGCTCACGCAGGGGCTTCAGTAGACCTGACCATTTTTTCTTTACACTTGGCAGGTGTCTCCTCAATTCTAGGGGCCATTAACTTTATTACAACAATTATCAATATAAAACCCCCTGCCATATCACAATATCAAACCCCTCTATTCGTGTGATCCGTATTAGTCACTGCTGTACTACTACTTCTCTCACTCCCTGTACTAGCAGCCGGAATCACAATACTATTAACAGACCGAAACCTAAATACAACCTTTTTTGACCCAGCAGGAGGCGGAGATCCTATTCTATATCAACACTTGTTC

RD01

TACTCTGTATCTATTATTTGGTGCCTGAGCAGGCATAGTAGGGACAGCCTTAAGCCTACTGATTCGTGCCGAACTGGGCCAACCTGGTACTCTACTTGGAGATGACCAAATTTATAATGTTATCGTAACCGCACATGCATTCGTAATAATTTTCTTTATAGTTATGCCAATTATAATTGGAGGATTTGGTAATTGACTAGTTCCCCTAATAATTGGCGCCCCAGACATAGCATTTCCTCGAATAAACAATATAAGCTTTTGACTCCTCCCTCCTTCTTTCTTACTACTCCTAGCATCATCTATAGTTGAAGCTGGCGCAGGAACAGGCTGAACTGTATATCCCCCTCTAGCTGGCAACTTAGCTCATGCAGGGGCTTCAGTAGACCTGACTATTTTTTCTTTACACTTAGCAGGCGTCTCCTCAATTCTAGGGGCCATTAACTTTATTACAACAATTATCAATATAAAACCCCCTGCCATATCACAATATCAAACCCCTCTATTTGTGTGATCCGTATTAGTCACTGCTGTACTACTACTTCTCTCACTCCCTGTACTAGCAGCCGGAATTACAATACTATTAACAGACCGAAACTTAAATACAACCTTCTTTGACCCAGCAGGAGGCGGAGATCCTATTCTATATCAACACTTGTTC

RD02

TACTCTGTATCTATTATTTGGTGCCTGAGCAGGCATAGTAGGGACAGCCTTAAGCCTACTGATTCGTGCCGAACTGGGCCAACCTGGTACTCTACTTGGAGATGACCAAATTTATAATGTTATCGTAACCGCACATGCATTCGTAATAATTTTCTTTATAGTTATGCCAATTATAATTGGAGGATTTGGTAATTGACTAGTTCCCCTAATAATTGGCGCCCCAGACATAGCATTTCCTCGAATAAACAATATAAGCTTTTGACTCCTCCCTCCTTCTTTCTTACTACTCCTAGCATCATCTATAGTTGAAGCTGGCGCAGGAACAGGCTGAACTGTATATCCCCCTCTAGCTGGCAACTTAGCTCATGCAGGGGCTTCAGTAGACCTGACTATTTTTTCTTTACACTTAGCAGGCGTCTCCTCAATTCTAGGGGCCATTAACTTTATTACAACAATTATCAATATAAAACCCCCTGCCATATCACAATATCAAACCCCTCTATTTGTGTGATCCGTATTAGTCACTGCTGTACTACTACTTCTCTCACTCCCTGTACTAGCAGCCGGAATTACAATACTATTAACAGACCGAAACTTAAATACAACCTTCTTTGACCCAGCAGGAGGCGGAGATCCTATTCTATATCAACACTTGTTC

**RD03**

TACTCTGTATCTATTATTTGGTGCCTGAGCAGGCATAGTAGGGACAGCCTTAAGCCTACTGATTCGTGCCGAACTGGGCCAACCTGGTACTCTACTTGGAGATGACCAAATTTATAATGTTATCGTAACCGCACATGCATTCGTAATAATTTTCTTTATAGTTATGCCAATTATAATTGGAGGATTTGGTAATTGACTAGTTCCCCTAATAATTGGCGCCCCAGACATAGCATTTCCTCGAATAAACAATATAAGCTTTTGACTCCTCCCTCCTTCTTTCTTACTACTCCTAGCATCATCTATAGTTGAAGCTGGCGCAGGAACAGGCTGAACTGTATATCCCCCTCTAGCTGGCAACTTAGCTCATGCAGGGGCTTCAGTAGACCTGACTATTTTTTCTTTACACTTAGCAGGCGTCTCCTCAATTCTAGGGGCCATTAACTTTATTACAACAATTATCAATATAAAACCCCCTGCCATATCACAATATCAAACCCCTCTATTTGTGTGATCCGTATTAGTCACTGCTGTACTACTACTTCTCTCACTCCCTGTACTAGCAGCCGGAATTACAATACTATTAACAGACCGAAACTTAAATACAACCTTCTTTGACCCAGCAGGAGGCGGAGATCCTATTCTATATCAACACTTGTTC

RD04

TACTCTGTATCTATTATTTGGTGCCTGAGCAGGCATAGTAGGGACAGCCTTAAGCCTACTGATTCGTGCCGAACTGGGCCAACCTGGTACTCTACTTGGAGATGACCAAATTTATAATGTTATCGTAACCGCACATGCATTCGTAATAATTTTCTTTATAGTTATGCCAATTATAATTGGAGGATTTGGTAATTGACTAGTTCCCCTAATAATTGGCGCCCCAGACATAGCATTTCCTCGAATAAACAATATAAGCTTTTGACTCCTCCCTCCTTCTTTCTTACTACTCCTAGCATCATCTATAGTTGAAGCTGGCGCAGGAACAGGCTGAACTGTATATCCCCCTCTAGCTGGCAACTTAGCTCATGCAGGGGCTTCAGTAGACCTGACTATTTTTTCTTTACACTTAGCAGGCGTCTCCTCAATTCTAGGGGCCATTAACTTTATTACAACAATTATCAATATAAAACCCCCTGCCATATCACAATATCAAACCCCTCTATTTGTGTGATCCGTATTAGTCACTGCTGTACTACTACTTCTCTCACTCCCTGTACTAGCAGCCGGAATTACAATACTATTAACAGACCGAAACTTAAATACAACCTTCTTTGACCCAGCAGGAGGCGGAGATCCTATTCTATATCAACACTTGTTC

RD05

TACTCTGTATCTACTATTTGGTGCCTGAGCAGGCATAGTAGGAACAGCCTTAAGCCTACTGATTCGTGCCGAACTGGGCCAACCTGGTACTCTGCTTGGAGATGATCAAATTTATAATGTTATCGTAACCGCACATGCATTCGTAATAATTTTCTTTATAGTTATACCAATTATAATCGGAGGATTTGGTAATTGACTAGTTCCCCTAATAATTGGTGCCCCAGACATAGCATTCCCTCGAATAAACAATATAAGCTTTTGACTCCTCCCTCCTTCTTTCTTACTACTTTTAGCATCATCTATAGTTGAAGCTGGCGCAGGAACAGGCTGAACTGTATATCCCCCTCTAGCTGGCAACTTAGCTCACGCAGGGGCTTCAGTAGACCTGACCATTTTTTCTTTACACTTGGCAGGTGTCTCCTCAATTCTAGGGGCCATTAACTTTATTACAACAATTATCAATATAAAACCCCCTGCCATATCACAATATCAAACCCCTCTATTCGTGTGATCCGTATTAGTCACTGCTGTACTACTACTTCTCTCACTCCCTGTACTAGCAGCCGGAATCACAATACTATTAACAGACCGAAACCTAAATACAACCTTTTTTGACCCAGCAGGAGGCGGAGATCCTATTCTATATCAACACTTGTTC

RD06

TACTCTGTATCTACTATTTGGTGCCTGAGCAGGCATAGTAGGAACAGCCTTAAGCCTACTGATTCGTGCCGAACTGGGCCAACCTGGTACTCTGCTTGGAGATGATCAAATTTATAATGTTATCGTAACCGCACATGCATTCGTAATAATTTTCTTTATAGTTATACCAATTATAATCGGAGGATTTGGTAATTGACTAGTTCCCCTAATAATTGGTGCCCCAGACATAGCATTCCCTCGAATAAACAATATAAGCTTTTGACTCCTCCCTCCTTCTTTCTTACTACTTTTAGCATCATCTATAGTTGAAGCTGGCGCAGGAACAGGCTGAACTGTATATCCCCCTCTAGCTGGCAACTTAGCTCACGCAGGGGCTTCAGTAGACCTGACCATTTTTTCTTTACACTTGGCAGGTGTCTCCTCAATTCTAGGGGCCATTAACTTTATTACAACAATTATCAATATAAAACCCCCTGCCATATCACAATATCAAACCCCTCTATTCGTGTGATCCGTATTAGTCACTGCTGTACTACTACTTCTCTCACTCCCTGTACTAGCAGCCGGAATCACAATACTATTAACAGACCGAAACCTAAATACAACCTTTTTTGACCCAGCAGGAGGCGGAGATCCTATTCTATATCAACACTTGTTC

RD07

TACTCTGTATCTACTATTTGGTGCCTGAGCAGGCATAGTAGGAACAGCCTTAAGCCTACTGATTCGTGCCGAACTGGGCCAACCTGGTACTCTGCTTGGAGATGATCAAATTTATAATGTTATCGTAACCGCACATGCATTCGTAATAATTTTCTTTATAGTTATACCAATTATAATCGGAGGATTTGGTAATTGACTAGTTCCCCTAATAATTGGTGCCCCAGACATAGCATTCCCTCGAATAAACAATATAAGCTTTTGACTCCTCCCTCCTTCTTTCTTACTACTTTTAGCATCATCTATAGTTGAAGCTGGCGCAGGAACAGGCTGAACTGTATATCCCCCTCTAGCTGGCAACTTAGCTCACGCAGGGGCTTCAGTAGACCTGACCATTTTTTCTTTACACTTGGCAGGTGTCTCCTCAATTCTAGGGGCCATTAACTTTATTACAACAATTATCAATATAAAACCCCCTGCCATATCACAATATCAAACCCCTCTATTCGTGTGATCCGTATTAGTCACTGCTGTACTACTACTTCTCTCACTCCCTGTACTAGCAGCCGGAATCACAATACTATTAACAGACCGAAACCTAAATACAACCTTTTTTGACCCAGCAGGAGGCGGAGATCCTATTCTATATCAACACTTGTTC

1. **Raw sequence of cyt b**

SD-ref (accession number OP866991)

TCATTCAACTACAAGAACACTAATGACCAATATCCGAAAAACCCACCCATTAATAAAAATTGTAAACAACGCATTCATTGACCTCCCCGCCCCATCAAATATTTCATCCTGATGAAATTTTGGCTCCTTACTAGGAATTTGTCTAATCCTACAAATTCTCACAGGCCTATTCCTAGCAATACACTATACATCTGACACAATAACAGCATTTTCCTCTGTCACCCATATCTGTCGAGATGTCAATTATGGTTGAATTATTCGATACATACACGCAAACGGGGCATCAATATTTTTCATCTGCCTATTCATACATGTAGGACGAGGCCTGTACTACGGATCATATACTTTTCTAGAGACATGGAACATCGGAGTAATCCTCCTATTTACAGTTATAGCCACAGCATTCGTAGGATAtGTCCTACCA

RD-ref (accession number OP866992)

TCATTCAACTACAAGAACACTAATGACCAATATCCGAAAAACCCACCCGCTAATAAAAATTGTAAACAACGCATTTATTGACCTCCCAGCCCCATCAAATATTTCATCCTGATGAAATTTCGGCTCATTACTAGGAATCTGTTTAATCCTACAAATCCTCACAGGCCTATTCCTAGCGATACACTATACATCTGATACAATAACAGCATTCTCCTCTGTCACCCATATCTGTCGAGATGTCAATTATGGCTGAATTATTCGATATATACACGCAAACGGGGCATCAATATTTTTCATCTGTCTATTCATACATGTAGGGCGAGGCCTGTACTACGGATCATATACTTTTCTAGAGACGTGAAACATCGGAGTAATTCTTCTATTTACAGTTATAGCCACAGCATTCGTAGGGTATGTCCTACCA

SD01

TCATTCAACTACAAGAACACTAATGACCAATATCCGAAAAACCCACCCATTAATAAAAATTGTAAACAACGCATTCATTGACCTCCCCGCCCCATCAAATATTTCATCCTGATGAAATTTTGGCTCCTTACTAGGAATTTGTCTAATCCTACAAATTCTCACAGGCCTATTCCTAGCAATACACTATACATCTGACACAATAACAGCATTTTCCTCTGTCACCCATATCTGTCGAGATGTCAATTATGGTTGAATTATTCGATACATACACGCAAACGGGGCATCAATATTTTTCATCTGCCTATTCATACATGTAGGACGAGGCCTGTACTACGGATCATATACTTTTCTAGAGACATGGAACATCGGAGTAATCCTCCTATTTACAGTTATAGCCACAGCATTCGTAGGATATGTCCTACCA

SD02

TCATTCAACTACAAGAACACTAATGACCAATATCCGAAAAACCCACCCATTAATAAAAATTGTAAACAACGCATTCATTGACCTCCCCGCCCCATCAAATATTTCATCCTGATGAAATTTTGGCTCCTTACTAGGAATTTGTCTAATCCTACAAATTCTCACAGGCCTATTCCTAGCAATACACTATACATCTGACACAATAACAGCATTTTCCTCTGTCACCCATATCTGTCGAGATGTCAATTATGGTTGAATTATTCGATACATACACGCAAACGGGGCATCAATATTTTTCATCTGCCTATTCATACATGTAGGACGAGGCCTGTACTACGGATCATATACTTTTCTAGAGACATGGAACATCGGAGTAATCCTCCTATTTACAGTTATAGCCACAGCATTCGTAGGATATGTCCTACCA

SD03

TCATTCAACTACAAGAACACTAATGACCAATATCCGAAAAACCCACCCATTAATAAAAATTGTAAACAACGCATTCATTGACCTCCCCGCCCCATCAAATATTTCATCCTGATGAAATTTTGGCTCCTTACTAGGAATTTGTCTAATCCTACAAATTCTCACAGGCCTATTCCTAGCAATACACTATACATCTGACACAATAACAGCATTTTCCTCTGTCACCCATATCTGTCGAGATGTCAATTATGGTTGAATTATTCGATACATACACGCAAACGGGGCATCAATATTTTTCATCTGCCTATTCATACATGTAGGACGAGGCCTGTACTACGGATCATATACTTTTCTAGAGACATGGAACATCGGAGTAATCCTCCTATTTACAGTTATAGCCACAGCATtCGTAGGATATGTCCTACCA

SD04

TCATTCAACTACAAGAACACTAATGACCAATATCCGAAAAACCCACCCATTAATAAAAATTGTAAACAACGCATTCATTGACCTCCCCGCCCCATCAAATATTTCATCCTGATGAAATTTTGGCTCCTTACTAGGAATTTGTCTAATCCTACAAATTCTCACAGGCCTATTCCTAGCAATACACTATACATCTGACACAATAACAGCATTTTCCTCTGTCACCCATATCTGTCGAGATGTCAATTATGGTTGAATTATTCGATACATACACGCAAACGGGGCATCAATATTTTTCATCTGCCTATTCATACATGTAGGACGAGGCCTGTACTACGGATCATATACTTTTCTAGAGACATGGAACATCGGAGTAATCCTCCTATTTACAGTTATAGCCACAGCATTCGTAGGATATGTCCTACCA

SD05

TCATTCAACTACAAGAACACTAATGATCAATATCCGAAAAACTCACCCATTGATAAAAATCGTAAACAACGCATTTATTGATCTCCCAGCCCCATCAAATATTTCATCCTGATGAAATTTTGGCTCCCTACTAGGAATTTGCTTAATCCTACAAATCCTCACAGGCCTATTCCTAGCAATACACTACACATCTGATACAATAACAGCATTTTCCTCTGTCACCCATATCTGCCGAGACGTCAATTACGGTTGAATCATTCGATACATGCACGCAAACGGAGCATCAATATTCTTTATCTGCCTATTCATCCATGTTGGACGAGGCCTATACTACGGATCATACACTTTTCTAGAGACATGGAACATCGGAGTAATTCTCCTATTTACAGTTATAGCTACAGCATtCGTAGGCTACGTCCTACCA

SD06

TCATTCAACTACAAGAACaCTAATGATCAATATCCGAAAAACTCACCCATTGATAAAAATCGTAAACAACGCATTTATTGATCTCCCAGCCCCATCAAATATTTCATCCTGATGAAATTTTGGCTCCCTACTAGGAATTTGCTTAATCCTACAAATCCTCACAGGCCTATTCCTAGCAATACACTACACATCTGATACAATAACAGCATTTTCCTCTGTCACCCATATCTGCCGAGACGTCAATTACGGTTGAATCATTCGATACATGCACGCAAACGGAGCATCAATATTCTTTATCTGCCTATTCATCCATGTTGGACGAGGCCTATACTACGGATCATACACTTTTCTAGAGACATGGAACATCGGAGTAATTCTCCTATTTACAGTTATAGCTACAGCATtCGTAGGCTACGTCCTACCA

SD07

TCATTCAACTACAAGAACACTAATGATCAATATCCGAAAAACTCACCCATTGATAAAAATCGTAAACAACGCATTTATTGATCTCCCAGCCCCATCAAATATTTCATCCTGATGAAATTTTGGCTCCCTACTAGGAATTTGCTTAATCCTACAAATCCTCACAGGCCTATTCCTAGCAATACACTACACATCTGATACAATAACAGCATTTTCCTCTGTCACCCATATCTGCCGAGACGTCAATTACGGTTGAATCATTCGATACATGCACGCAAACGGAGCATCAATATTCTTTATCTGCCTATTCATCCATGTTGGACGAGGCCTATACTACGGATCATACACTTTTCTAGAGACATGGAACATCGGAGTAATTCTCCTATTTACAGTTATAGCTACAGCATtCGTAGGCTACGTCCTACCA

SD08

TCATTCAACTACAAGAACACTAATGACCAATATCCGAAAAACCCACCCATTAATAAAAATTGTAAACAACGCATTCATTGACCTCCCCGCCCCATCAAATATTTCATCCTGATGAAATTTTGGCTCCTTACTAGGAATTTGTCTAATCCTACAAATTCTCACAGGCCTATTCCTAGCAATACACTATACATCTGACACAATAACAGCATTTTCCTCTGTCACCCATATCTGTCGAGATGTCAATTATGGTTGAATTATTCGATACATACACGCAAACGGGGCATCAATATTTTTCATCTGCCTATTCATACATGTAGGACGAGGCCTGTACTACGGATCATATACTTTTCTAGAGACATGGAACATCGGAGTAATCCTCCTATTTACAGTTATAGCCACAGCATTCGTAGGATATGTCCTACCA

SD09

TCATTCAACTATAAGAACACTAATGACCAATATCCGAAAAACCCACCCACTGATAAAAATTGTAAACAACGCATTTATTGACCTCCCAGCCCCATCAAATATTTCATCCTGATGAAATTTCGGCTCACTACTAGGAGTCTGTCTAATCCTACAAATCCTCACAGGCCTATTCCTAGCGATACACTATACATCTGATACAATAACAGCATTCTCCTCTGTCACCCATATCTGTCGAGATGTCAATTATGGCTGAATTATTCGATACATACACGCAAACGGGGCATCAATATTTTTCATCTGTCTGTTTATACATGTAGGACGAGGCCTGTACTACGGATCATATACTTTTCTAGAGACGTGAAATATCGGAGTAGTTCTTCTATTTACAGTTATAGCCACAGCATTCGTAGGGTATGTCCTACCA

SD10

TCATTCAACTACAAGAACATCAATGACCAACATCCGAAAAACCCACCCATTAATAAAAATTGTAAACAACGCATTTATTGACCTCCCAGCCCCATCAAACATCTCATCATGATGAAATTTTGGCTCTCTACTAGGAATCTGCTTAATTCTACAAATCCTTACCGGTCTATTTCTAGCAATACATTATACATCCGATACAATAACAGCATTCTCCTCTGTTACTCACATCTGTCGAGACGTCAATTATGGCTGAATCATCCGATACATACATGCCAACGGAGCATCAATATTTTTCATCTGCTTATTTATACATGTAGGACGAGGCCTATACTATGGATCATACACCTTCCTAGAAACATGAAATATTGGAGTGATCCTCTTATTTACAGTAATAGCTACAGCATTTGTAGGATATGTCCTACCA

SD11

TCATTCAACTACAAGAACACTAATGACCAATATCCGAAAAACCCACCCACTAATAAAAATTGTAAACAACGCATTTATTGACCTCCCAGCCCCATCAAATATTTCATCCTGATGAAATTTCGGCTCATTACTAGGAGTCTGTCTAATCCTACAAATCCTCACAGGCCTATTCCTAGCGATACACTATACATCTGATACAATAACAGCATTCTCCTCTGTCACCCATATCTGTCGAGATGTCAATTATGGCTGAATTATTCGATATATACACGCAAACGGGGCATCAATATTTTTCATCTGTCTATTCATACATGTAGGGCGAGGCCTGTACTACGGATCATATACTTTTCTAGAGACGTGAAACATCGGAGTAATTCTTCTATTTACAGTTATAGCCACAGCATTCGTAGGATATGTCCTACCA

SD12

TCATTCAACTACAAGAACACTAATGACCAATATCCGAAAAACCCACCCACTAATAAAAATTGTAAACAACGCATTTATTGACCTCCCAGCCCCATCAAATATTTCATCCTGATGAAATTTCGGCTCATTACTAGGARTCTGTCTAATCCTACAAATCCTCACAGGCCTATTCCTAGCGATACACTATACATCTGATACAATAACAGCATTCTCCTCTGTCACCCATATCTGTCGAGATGTCAATTATGGCTGAATTATTCGATATATACACGCAAACGGGGCATCAATATTTTTCATCTGTCTATTCATACATGTAGGGCGAGGCCTGTACTACGGATCATATACTTTTCTAGAGACGTGAAACATCGGAGTAGTTCTTCTATTTACAGTTATAGCCACAGCATTCGTAGGATATGTCCTACCA

SD13

TCATTCAACTACAAGAACACTAATGACCAATATCCGAAAAACCCACCCATTAATAAAAATTGTAAACAACGCATTCATTGACCTCCCCGCCCCATCAAATATTTCATCCTGATGAAATTTTGGCTCCTTACTAGGAATTTGTCTAATCCTACAAATTCTCACAGGCCTATTCCTAGCAATACACTATACATCTGACACAATAACAGCATTTTCCTCTGTCACCCATATCTGTCGAGATGTCAATTATGGTTGAATTATTCGATACATACACGCAAACGGGGCATCAATATTTTTCATCTGCCTATTCATACATGTAGGACGAGGCCTGTACTACGGATCATATACTTTTCTAGAGACATGGAACATCGGAGTAATCCTCCTATTTACAGTTATAGCCACAGCATTCGTAGGATATGTCCTACCA

SD14

TCATTCAACTACAAGAACACTAATGACCAATATCCGAAAAACCCACCCATTAATAAAAATTGTAAACAACGCATTCATTGACCTCCCCGCCCCATCAAATATTTCATCCTGATGAAATTTTGGCTCCTTACTAGGAATTTGTCTAATCCTACAAATTCTCACAGGCCTATTCCTAGCAATACACTATACATCTGACACAATAACAGCATTTTCCTCTGTCACCCATATCTGTCGAGATGTCAATTATGGTTGAATTATTCGATACATACACGCAAACGGGGCATCAATATTTTTCATCTGCCTATTCATACATGTAGGACGAGGCCTGTACTACGGATCATATACTTTTCTAGAGACATGGAACATCGGAGTAATCCTCCTATTTACAGTTATAGCCACAGCATTCGTAGGATATGTCCTACCA

SD15

TCATTCAACTACAAGAACACTAATGACCAATATCCGAAAAACCCACCCATTAATAAAAATTGTAAACAACGCATTCATTGACCTCCCCGCCCCATCAAATATTTCATCCTGATGAAATTTTGGCTCCTTACTAGGAATTTGTCTAATCCTACAAATTCTCACAGGCCTATTCCTAGCAATACACTATACATCTGACACAATAACAGCATTTTCCTCTGTCACCCATATCTGTCGAGATGTCAATTATGGTTGAATTATTCGATACATACACGCAAACGGGGCATCAATATTTTTCATCTGCCTATTCATACATGTAGGACGAGGCCTGTACTACGGATCATATACTTTTCTAGAGACATGGAACATCGGAGTAATCCTCCTATTTACAGTTATAGCCACAGCATtCGTAGGATATGTCCTACCA

RD01

TCATTCAACTACAAGAACACTAATGACCAATATCCGAAAAACCCACCCACTAATAAAAATTGTAAACAACGCATTTATTGACCTCCCAGCCCCATCAAATATTTCATCCTGATGAAATTTCGGCTCATTACTAGGAGTCTGCCTAATCCTACAAATCCTCACAGGCCTATTCCTAGCGATACACTATACATCTGATACAATAACAGCATTCTCCTCTGTCACCCATATCTGTCGAGATGTCAATTATGGCTGAATTATTCGATATATACACGCAAACGGGGCATCAATATTTTTCATCTGTCTATTCATACATGTAGGGCGAGGCCTGTACTACGGATCATATACTTTTCTAGAGACGTGAAACATCGGAGTAGTTCTTCTATTTACAGTTATAGCCACAGCATTCGTAGGATATGTCCTACCA

RD02

TCATTCAACTACAAGAACACTAATGACCAATATCCGAAAAACCCACCCACTAATAAAAATTGTAAACAACGCATTTATTGACCTCCCAGCCCCATCAAATATTTCATCCTGATGAAATTTCGGCTCATTACTAGGAGTCTGCCTAATCCTACAAATCCTCACAGGCCTATTCCTAGCGATACACTATACATCTGATACAATAACAGCATTCTCCTCTGTCACCCATATCTGTCGAGATGTCAATTATGGCTGAATTATTCGATATATACACGCAAACGGGGCATCAATATTTTTCATCTGTCTATTCATACATGTAGGGCGAGGCCTGTACTACGGATCATATACTTTTCTAGAGACGTGAAACATCGGAGTAGTTCTTCTATTTACAGTTATAGCCACAGCATtCGTAGGATATGTCCTACCA

RD03

TCATTCAACTACAAGAACACTAATGACCAATATCCGAAAAACCCACCCACTAATAAAAATTGTAAACAACGCATTTATTGACCTCCCAGCCCCATCAAATATTTCATCCTGATGAAATTTCGGCTCATTACTAGGAGTCTGCCTAATCCTACAAATCCTCACAGGCCTATTCCTAGCGATACACTATACATCTGATACAATAACAGCATTCTCCTCTGTCACCCATATCTGTCGAGATGTCAATTATGGCTGAATTATTCGATATATACACGCAAACGGGGCATCAATATTTTTCATCTGTCTATTCATACATGTAGGGCGAGGCCTGTACTACGGATCATATACTTTTCTAGAGACGTGAAACATCGGAGTAGTTCTTCTATTTACAGTTATAGCCACAGCATTCGTAGGATATGTCCTACCA

RD04

TCATTCAACTACAAGAACACTAATGACCAATATCCGAAAAACCCACCCACTAATAAAAATTGTAAACAACGCATTTATTGACCTCCCAGCCCCATCAAATATTTCATCCTGATGAAATTTCGGCTCATTACTAGGAGTCTGCCTAATCCTACAAATCCTCACAGGCCTATTCCTAGCGATACACTATACATCTGATACAATAACAGCATTCTCCTCTGTCACCCATATCTGTCGAGATGTCAATTATGGCTGAATTATTCGATATATACACGCAAACGGGGCATCAATATTTTTCATCTGTCTATTCATACATGTAGGGCGAGGCCTGTACTACGGATCATATACTTTTCTAGAGACGTGAAACATCGGAGTAGTTCTTCTATTTACAGTTATAGCCACAGCATTCGTAGGATATGTCCTACCA

RD05

TCATTCAACTACAAGAACACTAATGACCAATATCCGAAAAACCCACCCATTAATAAAAATTGTAAACAACGCATTCATTGACCTCCCCGCCCCATCAAATATTTCATCCTGATGAAATTTTGGCTCCTTACTAGGAATTTGTCTAATCCTACAAATTCTCACAGGCCTATTCCTAGCAATACACTATACATCTGACACAATAACAGCATTTTCCTCTGTCACCCATATCTGTCGAGATGTCAATTATGGTTGAATTATTCGATACATACACGCAAACGGGGCATCAATATTTTTCATCTGCCTATTCATACATGTAGGACGAGGCCTGTACTACGGATCATATACTTTTCTAGAGACATGGAACATCGGAGTAATCCTCCTATTTACAGTTATAGCCACAGCATtCGTAGGATATGTCCTACCA

RD06

TCATTCAACTACAAGAACACTAATGACCAATATCCGAAAAACCCACCCATTAATAAAAATTGTAAACAACGCATTCATTGACCTCCCCGCCCCATCAAATATTTCATCCTGATGAAATTTTGGCTCCTTACTAGGAATTTGTCTAATCCTACAAATTCTCACAGGCCTATTCCTAGCAATACACTATACATCTGACACAATAACAGCATTTTCCTCTGTCACCCATATCTGTCGAGATGTCAATTATGGTTGAATTATTCGATACATACACGCAAACGGGGCATCAATATTTTTCATCTGCCTATTCATACATGTAGGACGAGGCCTGTACTACGGATCATATACTTTTCTAGAGACATGGAACATCGGAGTAATCCTCCTATTTACAGTTATAGCCACAGCATtCGTAGGATATGTCCTACCA

RD07

TCATTCAACTACAAGAACACTAATGACCAATATCCGAAAAACCCACCCATTAATAAAAATTGTAAACAACGCATTCATTGACCTCCCCGCCCCATCAAATATTTCATCCTGATGAAATTTTGGCTCCTTACTAGGAATTTGTCTAATCCTACAAATTCTCACAGGCCTATTCCTAGCAATACACTATACATCTGACACAATAACAGCATTTTCCTCTGTCACCCATATCTGTCGAGATGTCAATTATGGTTGAATTATTCGATACATACACGCAAACGGGGCATCAATATTTTTCATCTGCCTATTCATACATGTAGGACGAGGCCTGTACTACGGATCATATACTTTTCTAGAGACATGGAACATCGGAGTAATCCTCCTATTTACAGTTATAGCCACAGCATtCGTAGGATATGTCCTACCA

1. **Raw sequence of rrn12**

SD-ref (accession number OP856556)

GCCTAGCCTTAaaCaCAAATAGTTATGTAAACAAAACTATTCGCCAGAGTACTACCGGCAATAGCTTAAAACTCAAAGGACTTGGCGGTGCTTTATACCCTTCTAGAGGAGCCTGTTCTATAATCGATAAACCCCGATAAACCTCACCATTCCTTGCTACTACAGTCTATATACCGCCATCTTCAGCAAACCCTAAAAAGGTACAAAAGTAAGCACAATCATAATACATAAAAACGTTAGGTCAAGGTGTAACCTATGGAACGGAAAGAAATGGGCTACATTTTCTAATCTAAGAAAATCCAACACGAAAGTTATTATGAAACTAATAACCAAAGGAGGATTTAGCAGTAAACTAAGAATAGAGTGCTTAGTtGAaTtAGgCCATGAAGCACGC

RD-ref (accession number OP856557)

GCCTAGCCTTAAACACAAATAGTTATGCAAACAAAACTATTCGCCAGAGTACTACCGGCAATAGCTTAAAACTCAAAGGACTTGGCGGTGCTTTACACCCTTCTAGAGGAGCCTGTTCTATAATCGATAAACCCCGATAAACCTCACCATTCCTTGCTAATACAGTCTATATACCGCCATCTTCAGCGAACCCTAAAAAGGTACAAAAGTAAGCACAATCATAATACATAAAGACGTTAGGTCAAGGTGTAACCTATGGAACGGAAAGAAATGGGCTACATTTTCTAATCTAAGAAAATCCAATACGAAAGTTATTATGAAATTAATAACCAAAGGAGGATTTAGCAGTAAACTAAGAATAGAGTGCTTAGTTGAACTAGGCCATGAAGCACGC

SD01

GCCTAGCCTtAAACACAAATAGTTATGTAAACAAAACTATTCGCCAGAGTACTACCGGCAATAGCTTAAAACTCAAAGGACTTGGCGGTGCTTTATACCCTTCTAGAGGAGCCTGTTCTATAATCGATAAACCCCGATAAACCTCACCATTCCTTGCTACTACAGTCTATATACCGCCATCTTCAGCAAACCCTAAAAAGGTACAAAAGTAAGCACAATCATAATACATAAAAACGTTAGGTCAAGGTGTAACCTATGGAACGGAAAGAAATGGGCTACATTTTCTAATCTAAGAAAATCCAACACGAAAGTTATTATGAAACTAATAACCAAAGGAGGATTTAGCAGTAAACTAAGAATAGAGTGCTTAGTTGAATTAGGCCATGAAGCACGC

SD02

GCCTAGCCTTAAACACAAATAGTTATGTAAACAAAACTATTCGCCAGAGTACTACCGGCAATAGCTTAAAACTCAAAGGACTTGGCGGTGCTTTATACCCTTCTAGAGGAGCCTGTTCTATAATCGATAAACCCCGATAAACCTCACCATTCCTTGCTACTACAGTCTATATACCGCCATCTTCAGCAAACCCTAAAAAGGTACAAAAGTAAGCACAATCATAATACATAAAAACGTTAGGTCAAGGTGTAACCTATGGAACGGAAAGAAATGGGCTACATTTTCTAATCTAAGAAAATCCAACACGAAAGTTATTATGAAACTAATAACCAAAGGAGGATTTAGCAGTAAACTAAGAATAGAGTGCTTAGTTGAATTAGGCCATGAAGCACGC

SD03

GCCTAGCCTTAAACACAAATAGTTATGTAAACAAAACTATTCGCCAGAGTACTACCGGCAATAGCTTAAAACTCAAAGGACTTGGCGGTGCTTTATACCCTTCTAGAGGAGCCTGTTCTATAATCGATAAACCCCGATAAACCTCACCATTCCTTGCTACTACAGTCTATATACCGCCATCTTCAGCAAACCCTAAAAAGGTACAAAAGTAAGCACAATCATAATACATAAAAACGTTAGGTCAAGGTGTAACCTATGGAACGGAAAGAAATGGGCTACATTTTCTAATCTAAGAAAATCCAACACGAAAGTTATTATGAAACTAATAACCAAAGGAGGATTTAGCAGTAAACTAAGAATAGAGTGCTTAGTTGAATTAGGCCATGAAGCACGC

SD04

GCCTAGCCTTAAACACAAATAGTTATGTAAACAAAACTATTCGCCAGAGTACTACCGGCAATAGCTTAAAACTCAAAGGACTTGGCGGTGCTTTATACCCTTCTAGAGGAGCCTGTTCTATAATCGATAAACCCCGATAAACCTCACCATTCCTTGCTACTACAGTCTATATACCGCCATCTTCAGCAAACCCTAAAAAGGTACAAAAGTAAGCACAATCATAATACATAAAAACGTTAGGTCAAGGTGTAACCTATGGAACGGAAAGAAATGGGCTACATTTTCTAATCTAAGAAAATCCAACACGAAAGTTATTATGAAACTAATAACCAAAGGAGGATTTAGCAGTAAACTAAGAATAGAGTGCTTAGTTGAATTAGGCCATGAAGCACGC

SD05

GCCTAGCCCTAAACACAAATAGTTGTGTAAACAAAACTATTCGCCAGAGTACTACCGGCAATAGCTTAAAACTCAAAGGACTTGGCGGTGCTTTATACCCTTCTAGAGGAGCCTGTTCTATAATCGATAAACCCCGATAAACCTCACCATTCCTTGCTAATACAGTCTATATACCGCCACCTTCAGCAAACCCTAAAAAGGTACAAAAGTAAGCACAATCATAATACATAAAAACGTTAGGTCAAGGTGTAACCTATGGAATGGGAAGAAATGGGCTACATTTTCTAACCTAAGAAAATCTAATACGAAAGTTATTATGAAACCAGTAACCAAAGGAGGATTTAGCAGTAAACTAAGAATAGAGTGCTTAGTTGAATTAGGCCATGAAGCACGC

SD06

GCCTAGCCCTAAACACAAATAGTTGTGTAAACAAAACTATTCGCCAGAGTACTACCGGCAATAGCTTAAAACTCAAAGGACTTGGCGGTGCTTTATACCCTTCTAGAGGAGCCTGTTCTATAATCGATAAACCCCGATAAACCTCACCATTCCTTGCTAATACAGTCTATATACCGCCACCTTCAGCAAACCCTAAAAAGGTACAAAAGTAAGCACAATCATAATACATAAAAACGTTAGGTCAAGGTGTAACCTATGGAATGGGAAGAAATGGGCTACATTTTCTAACCTAAGAAAATCTAATACGAAAGTTATTATGAAACCAGTAACCAAAGGAGGATTTAGCAGTAAACTAAGAATAGAGTGCTTAGTTGAATTAGGCCATGAAGCACGC

SD07

GCCTAGCCCTAAACACAAATAGTTGTGTAAACAAAACTATTCGCCAGAGTACTACCGGCAATAGCTTAAAACTCAAAGGACTTGGCGGTGCTTTATACCCTTCTAGAGGAGCCTGTTCTATAATCGATAAACCCCGATAAACCTCACCATTCCTTGCTAATACAGTCTATATACCGCCACCTTCAGCAAACCCTAAAAAGGTACAAAAGTAAGCACAATCATAATACATAAAAACGTTAGGTCAAGGTGTAACCTATGGAATGGGAAGAAATGGGCTACATTTTCTAACCTAAGAAAATCTAATACGAAAGTTATTATGAAACCAGTAACCAAAGGAGGATTTAGCAGTAAACTAAGAATAGAGTGCTTAGTTGAATTAGGCCATGAAGCACGC

SD08

GCCTAGCCTTAAACACAAATAGTTATGTAAACAAAACTATTCGCCAGAGTACTACCGGCAATAGCTTAAAACTCAAAGGACTTGGCGGTGCTTTATACCCTTCTAGAGGAGCCTGTTCTATAATCGATAAACCCCGATAAACCTCACCATTCCTTGCTACTACAGTCTATATACCGCCATCTTCAGCAAACCCTAAAAAGGTACAAAAGTAAGCACAATCATAATACATAAAAACGTTAGGTCAAGGTGTAACCTATGGAACGGAAAGAAATGGGCTACATTTTCTAATCTAAGAAAATCCAACACGAAAGTTATTATGAAACTAATAACCAAAGGAGGATTTAGCAGTAAACTAAGAATAGAGTGCTTAGTTGAATTAGGCCATGAAGCACGC

SD09

GCCTAGCCTTAAACACAAATAGTTATGCAAACAAAACTATTCGCCAGAGTACTACCGGCAATAGCTTAAAACTCAAAGGACTTGGCGGTGCTTTATACCCTTCTAGAGGAGCCTGTTCTATAATCGATAAACCCCGATAAACCTCACCATTCCTTGCTAATACAGTCTATATACCGCCATCTTCAGCGAACCCTAAAAAGGTACAAAAGTAAGCACAATCATAATACATAAAGACGTTAGGTCAAGGTGTAACCTATGGAACGGAAAGAAATGGGCTACATTTTCTAATCTAAGAAAATCCAACACGAAAGTTATTATGAAATTAATAACCAAAGGAGGATTTAGCAGTAAACTAAGAATAGAGTGCTTAGTTGAACTAGGCCATGAAGCACGC

SD10

GCTTAGCCCTAAACACAAGTAGTTATATAAACAAAACTATTCGCCAGAGTACTACCGGCAATAGCTTAAAACTCAAAGGACTTGGCGGTGCTTTATACCCTTCTAGAGGAGCCTGTTCTATAATCGATAAACCCCGATAAACCTCACCACCCCTTGCTAATACAGTCTATATACCGCCATCTTCAGCAAACCCTAAAAAGGAACAAAAGTAAGCACAATCATCATACGTAAAAACGTTAGGTCAAGGTGTAACCTATGGAGTGGAAAGAAATGGGCTACATTTTCTAACTTAAGAAAACCCCCTACGAAAGTTATTATGAAATTAGTAACCAAAGGAGGATTTAGCAGTAAACTAAGAATAGAGTGCTTAGTTGAATTAGGCCATGAAGCACGC

SD11

GCCTAGCCTTAAACACAAATAGTTATGCAAACAAAACTATTCGCCAGAGTACTACCGGCAATAGCTTAAAACTCAAAGGACTTGGCGGTGCTTTATACCCTTCTAGAGGAGCCTGTTCTATAATCGATAAACCCCGATAAACCTCACCATTCCTTGCTAATACAGTCTATATACCGCCATCTTCAGCGAACCCTAAAAAGGTACAAAAGTAAGCACAATCATAATACATAAAGACGTTAGGTCAAGGTGTAACCTATGGAACGGAAAGAAATGGGCTACATTTTCTAATCTAAGAAAATCCAACACGAAAGTTATTATGAAATTAATAACCAAAGGAGGATTTAGCAGTAAACTAAGAATAGAGTGCTTAGTTGAACTAGGCCATGAAGCACGC

SD12

GCCTAGCCTTAAACACAAATAGTTATGCAAACAAAACTATTCGCCAGAGTACTACCGGCAATAGCTTAAAACTCAAAGGACTTGGCGGTGCTTTATACCCTTCTAGAGGAGCCTGTTCTATAATCGATAAACCCCGATAAACCTCACCATTCCTTGCTAATACAGTCTATATACCGCCATCTTCAGCGAACCCTAAAAAGGTACAAAAGTAAGCACAATCATAATACATAAAGACGTTAGGTCAAGGTGTAACCTATGGAACGGAAAGAAATGGGCTACATTTTCTAATCTAAGAAAATCCAACACGAAAGTTATTATGAAATTAATAACCAAAGGAGGATTTAGCAGTAAACTAAGAATAGAGTGCTTAGTTGAACTAGGCCATGAAGCACGC

SD13

GCCTAGCCTTAAACACAAATAGTTATGTAAACAAAACTATTCGCCAGAGTACTACCGGCAATAGCTTAAAACTCAAAGGACTTGGCGGTGCTTTATACCCTTCTAGAGGAGCCTGTTCTATAATCGATAAACCCCGATAAACCTCACCATTCCTTGCTACTACAGTCTATATACCGCCATCTTCAGCAAACCCTAAAAAGGTACAAAAGTAAGCACAATCATAATACATAAAAACGTTAGGTCAAGGTGTAACCTATGGAACGGAAAGAAATGGGCTACATTTTCTAATCTAAGAAAATCCAACACGAAAGTTATTATGAAACTAATAACCAAAGGAGGATTTAGCAGTAAACTAAGAATAGAGTGCTTAGTTGAATTAGGCCATGAAGCACGC

SD14

GCCTAGCCTTAAACACAAATAGTTATGTAAACAAAACTATTCGCCAGAGTACTACCGGCAATAGCTTAAAACTCAAAGGACTTGGCGGTGCTTTATACCCTTCTAGAGGAGCCTGTTCTATAATCGATAAACCCCGATAAACCTCACCATTCCTTGCTACTACAGTCTATATACCGCCATCTTCAGCAAACCCTAAAAAGGTACAAAAGTAAGCACAATCATAATACATAAAAACGTTAGGTCAAGGTGTAACCTATGGAACGGAAAGAAATGGGCTACATTTTCTAATCTAAGAAAATCCAACACGAAAGTTATTATGAAACTAATAACCAAAGGAGGATTTAGCAGTAAACTAAGAATAGAGTGCTTAGTTGAATTAGGCCATGAAGCACGC

SD15

GCCTAGCCTTAAACACAAATAGTTATGTAAACAAAACTATTCGCCAGAGTACTACCGGCAATAGCTTAAAACTCAAAGGACTTGGCGGTGCTTTATACCCTTCTAGAGGAGCCTGTTCTATAATCGATAAACCCCGATAAACCTCACCATTCCTTGCTACTACAGTCTATATACCGCCATCTTCAGCAAACCCTAAAAAGGTACAAAAGTAAGCACAATCATAATACATAAAAACGTTAGGTCAAGGTGTAACCTATGGAACGGAAAGAAATGGGCTACATTTTCTAATCTAAGAAAATCCAACACGAAAGTTATTATGAAACTAATAACCAAAGGAGGATTTAGCAGTAAACTAAGAATAGAGTGCTTAGTTGAATTAGGCCATGAAGCACGC

RD01

GCCTAGCCTTAAACACAAATAGTTATGCAAACAAAACTATTCGCCAGAGTACTACCGGCAATAGCTTAAAACTCAAAGGACTTGGCGGTGCTTTATACCCTTCTAGAGGAGCCTGTTCTATAATCGATAAACCCCGATAAACCTCACCATTCCTTGCTAATACAGTCTATATACCGCCATCTTCAGCGAACCCTAAAAAGGTACAAAAGTAAGCACAATCATAATACATAAAGACGTTAGGTCAAGGTGTAACCTATGGAACGGAAAGAAATGGGCTACATTTTCTAATCTAAGAAAATCCAACACGAAAGTTATTATGAAATTAATAACCAAAGGAGGATTTAGCAGTAAACTAAGAATAGAGTGCTTAGTTGAACTAGGCCATGAAGCACGC

RD02

GCCTAGCCTTAAACACAAATAGTTATGCAAACAAAACTATTCGCCAGAGTACTACCGGCAATAGCTTAAAACTCAAAGGACTTGGCGGTGCTTTATACCCTTCTAGAGGAGCCTGTTCTATAATCGATAAACCCCGATAAACCTCACCATTCCTTGCTAATACAGTCTATATACCGCCATCTTCAGCGAACCCTAAAAAGGTACAAAAGTAAGCACAATCATAATACATAAAGACGTTAGGTCAAGGTGTAACCTATGGAACGGAAAGAAATGGGCTACATTTTCTAATCTAAGAAAATCCAACACGAAAGTTATTATGAAATTAATAACCAAAGGAGGATTTAGCAGTAAACTAAGAATAGAGTGCTTAGTTGAACTAGGCCATGAAGCACGC

RD03

GCCTAGCCTTAAACACAAATAGTTATGCAAACAAAACTATTCGCCAGAGTACTACCGGCAATAGCTTAAAACTCAAAGGACTTGGCGGTGCTTTATACCCTTCTAGAGGAGCCTGTTCTATAATCGATAAACCCCGATAAACCTCACCATTCCTTGCTAATACAGTCTATATACCGCCATCTTCAGCGAACCCTAAAAAGGTACAAAAGTAAGCACAATCATAATACATAAAGACGTTAGGTCAAGGTGTAACCTATGGAACGGAAAGAAATGGGCTACATTTTCTAATCTAAGAAAATCCAACACGAAAGTTATTATGAAATTAATAACCAAAGGAGGATTTAGCAGTAAACTAAGAATAGAGTGCTTAGTtGAACTAGGCCATGAAGCACGC

RD04

GCCTAGCCTTAAACACAAATAGTTATGCAAACAAAACTATTCGCCAGAGTACTACCGGCAATAGCTTAAAACTCAAAGGACTTGGCGGTGCTTTATACCCTTCTAGAGGAGCCTGTTCTATAATCGATAAACCCCGATAAACCTCACCATTCCTTGCTAATACAGTCTATATACCGCCATCTTCAGCGAACCCTAAAAAGGTACAAAAGTAAGCACAATCATAATACATAAAGACGTTAGGTCAAGGTGTAACCTATGGAACGGAAAGAAATGGGCTACATTTTCTAATCTAAGAAAATCCAACACGAAAGTTATTATGAAATTAATAACCAAAGGAGGATTTAGCAGTAAACTAAGAATAGAGTGCTTAGTTGAACTAGGCCATGAAGCACGC

RD05

GCCTAGCCTTAAACACAAATAGTTATGTAAACAAAACTATTCGCCAGAGTACTACCGGCAATAGCTTAAAACTCAAAGGACTTGGCGGTGCTTTATACCCTTCTAGAGGAGCCTGTTCTATAATCGATAAACCCCGATAAACCTCACCATTCCTTGCTACTACAGTCTATATACCGCCATCTTCAGCAAACCCTAAAAAGGTACAAAAGTAAGCACAATCATAATACATAAAAACGTTAGGTCAAGGTGTAACCTATGGAACGGAAAGAAATGGGCTACATTTTCTAATCTAAGAAAATCCAACACGAAAGTTATTATGAAACTAATAACCAAAGGAGGATTTAGCAGTAAACTAAGAATAGAGTGCTTAGTTGAATTAGGCCATGAAGCACGC

RD06

GCCTAGCCTTAAACACAAATAGTTATGTAAACAAAACTATTCGCCAGAGTACTACCGGCAATAGCTTAAAACTCAAAGGACTTGGCGGTGCTTTATACCCTTCTAGAGGAGCCTGTTCTATAATCGATAAACCCCGATAAACCTCACCATTCCTTGCTACTACAGTCTATATACCGCCATCTTCAGCAAACCCTAAAAAGGTACAAAAGTAAGCACAATCATAATACATAAAAACGTTAGGTCAAGGTGTAACCTATGGAACGGAAAGAAATGGGCTACATTTTCTAATCTAAGAAAATCCAACACGAAAGTTATTATGAAACTAATAACCAAAGGAGGATTTAGCAGTAAACTAAGAATAGAGTGCTTAGTTGAATTAGGCCATGAAGCACGC

RD07

GCCTAGCCTTAAACACAAATAGTTATGTAAACAAAACTATTCGCCAGAGTACTACCGGCAATAGCTTAAAACTCAAAGGACTTGGCGGTGCTTTATACCCTTCTAGAGGAGCCTGTTCTATAATCGATAAACCCCGATAAACCTCACCATTCCTTGCTACTACAGTCTATATACCGCCATCTTCAGCAAACCCTAAAAAGGTACAAAAGTAAGCACAATCATAATACATAAAAACGTTAGGTCAAGGTGTAACCTATGGAACGGAAAGAAATGGGCTACATTTTCTAATCTAAGAAAATCCAACACGAAAGTTATTATGAAACTAATAACCAAAGGAGGATTTAGCAGTAAACTAAGAATAGAGTGCTTAGTTGAATTAGGCCATGAAGCACGC
